# Supplementary material for: A genome‐wide RNAi screen reveals essential therapeutic targets of breast cancer stem cells
Source: EMBO Mol Med. 2019 Sep 2;11(10):e9930. doi: 10.15252/emmm.201809930 (PMC6783652; doi:10.15252/emmm.201809930)
Supplement: Supplementary file 1 — Appendix [file EMMM-11-e9930-s001.pdf]

Appendix

Table of contents

Appendix Figure S1

Appendix Figure S2

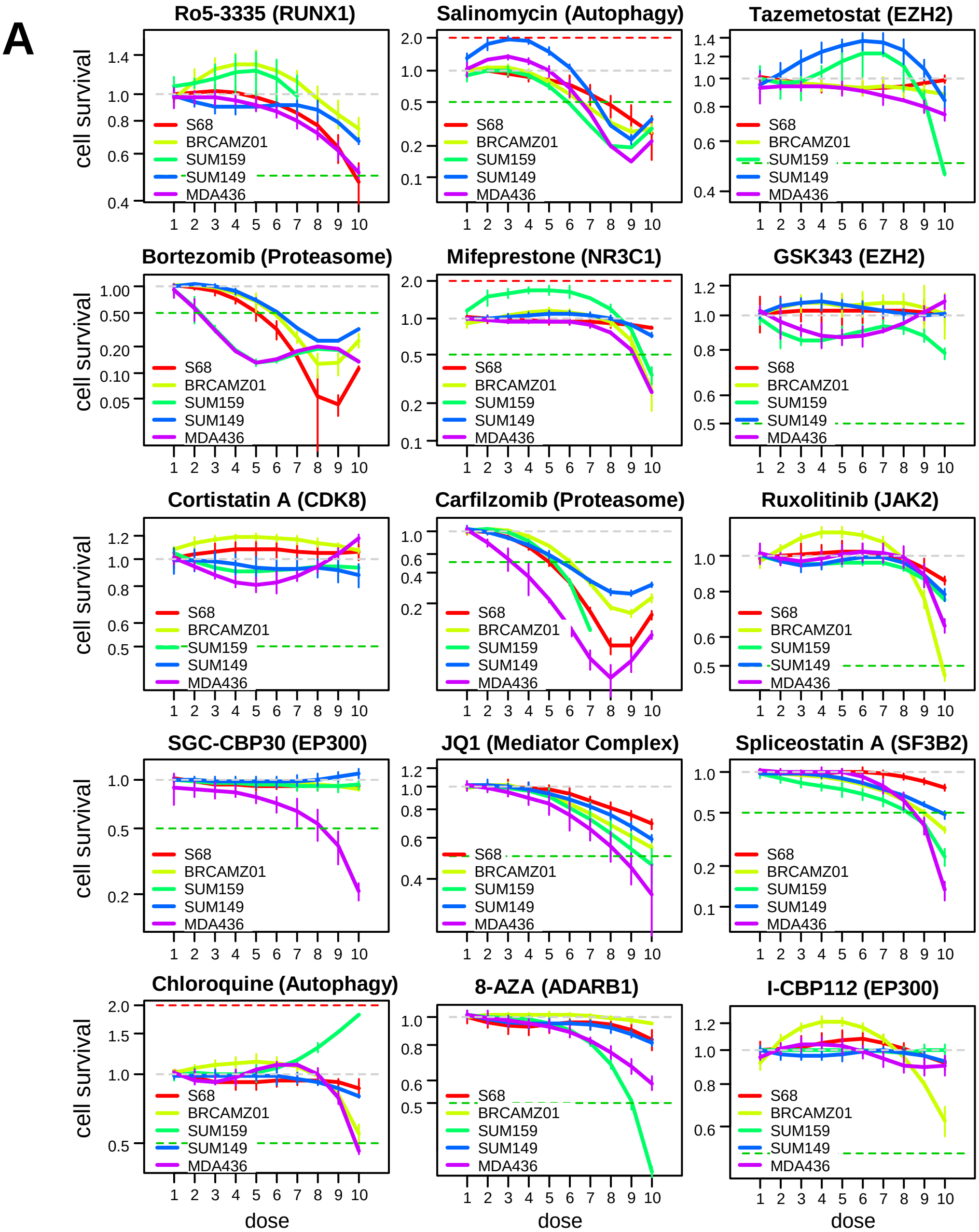

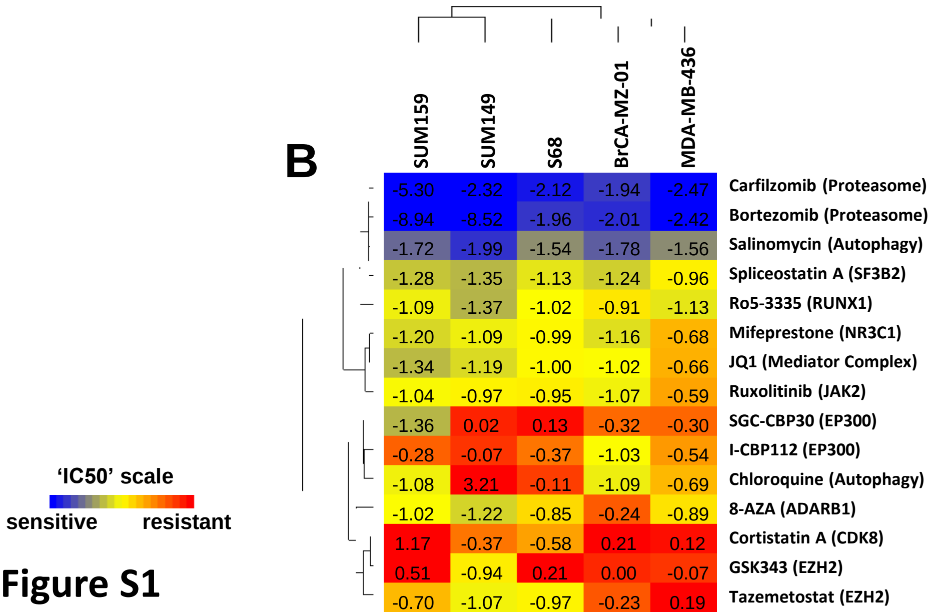

	SUM159	SUM149	S68	BrCA-MZ-01	MDA-MB-436
Carfilzomib (Proteasome)	-5.30	-2.32	-2.12	-1.94	-2.47
Bortezomib (Proteasome)	-8.94	-8.52	-1.96	-2.01	-2.42
Salinomycin (Autophagy)	-1.72	-1.99	-1.54	-1.78	-1.56
Spliceostatin A (SF3B2)	-1.28	-1.35	-1.13	-1.24	-0.96
Ro5-3335 (RUNX1)	-1.09	-1.37	-1.02	-0.91	-1.13
Mifeprestone (NR3C1)	-1.20	-1.09	-0.99	-1.16	-0.68
JQ1 (Mediator Complex)	-1.34	-1.19	-1.00	-1.02	-0.66
Ruxolitinib (JAK2)	-1.04	-0.97	-0.95	-1.07	-0.59
SGC-CBP30 (EP300)	-1.36	0.02	0.13	-0.32	-0.30
I-CBP112 (EP300)	-0.28	-0.07	-0.37	-1.03	-0.54
Chloroquine (Autophagy)	-1.08	3.21	-0.11	-1.09	-0.69
8-AZA (ADARB1)	-1.02	-1.22	-0.85	-0.24	-0.89
Cortistatin A (CDK8)	1.17	-0.37	-0.58	0.21	0.12
GSK343 (EZH2)	0.51	-0.94	0.21	0.00	-0.07
Tazemetostat (EZH2)	-0.70	-1.07	-0.97	-0.23	0.19

Appendix Figure S1

Appendix Figure S1. Dose curves of cell viability in breast cancer cell lines (BCLs). A. Dose curves representing the viability of five BCLs (BrCa-MZ-01, MDA-MB-436, S68, SUM149, SUM159) following treatment with the 15 drugs of the panel. Mean±SEM, n=4. The green dashed-line corresponds to a reduction of 50% of cell viability in the treated conditions compared to the control (grey dashed-line) and an increase of 50% of cell viability for the red dashed-line **B.** Heat map representing the IC50 for the drug panel, in five different cell lines. IC50 for each drugs/BCLs were plotted ranging from red (Resistant, IC50 not reached) to blue (Sensitive, lowest IC50).

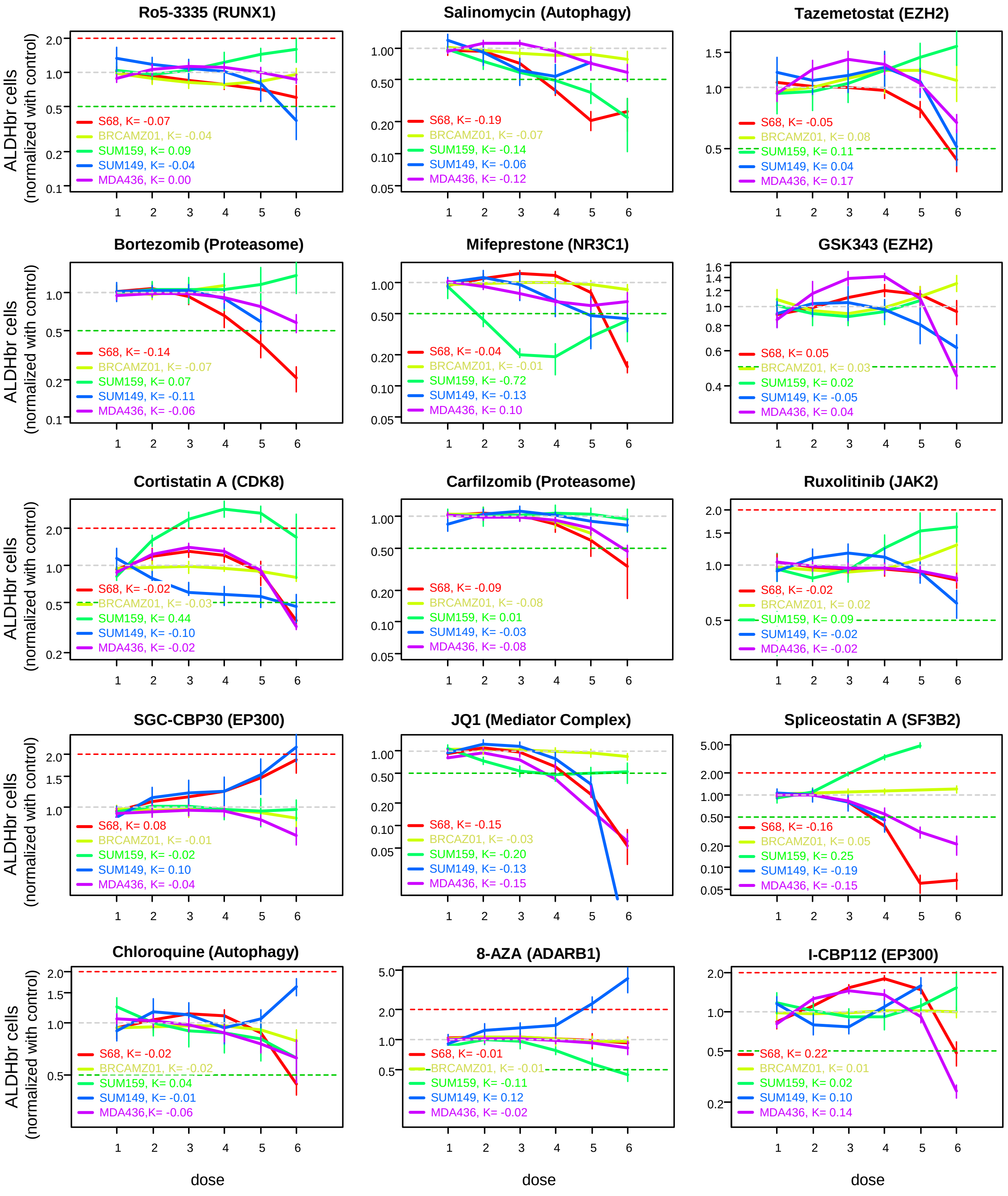

Appendix Figure S2

Appendix Figure S2. Dose curves of ALDHbr cell proportion in breast cancer cell lines (BCLs). Dose curves representing the variation of the ALDHbr cell proportion under treatment compared to the untreated condition, in five BCLs (BrCa-MZ-01, MDA-MB-436, S68, SUM149, SUM159). The green dashed-line corresponds to a reduction of 50% of the ALDHbr cell proportion in the treated conditions compared to the control (grey dashed-line) and an increase of 50% of the ALDHbr cell proportion for the red dashed-line. Mean±SEM, n=4.
