# Supplementary material for: A genome‐wide RNAi screen reveals essential therapeutic targets of breast cancer stem cells
Source: EMBO Mol Med. 2019 Sep 2;11(10):e9930. doi: 10.15252/emmm.201809930 (PMC6783652; doi:10.15252/emmm.201809930)
Supplement: Supplementary file 2 — Expanded View Figures PDF [file EMMM-11-e9930-s002.pdf]

Expanded View Figures

Figure EV1. Drug combination screening results.

A, B Interaction surface built using the estimated combination index (CI) for the mifepristone/salinomycin (A) or JQ1/mifepristone (B) drug combination, in four different breast cancer cell lines (top panels). The CI is represented in 2D color code interaction surface. The color code spans from strong synergism (dark blue, CI < 1) to strong antagonism (red, CI > 1). The concentrations of each particular drug in the combinations are denoted on each axis. On the bottom panels, neighborhood *Z*-core matrix estimating the significant synergistic interaction. The color code spans from a statistically significant synergism (dark blue, CI < 1, $P < 0.01$) to a statistically significant antagonism (dark red, CI > 1, $P < 0.01$).

C Violin plot representing the relative proportion of ALDHbr cells in SUM159 following gene silencing combinations. For each condition, a siRNA pool (3 siRNAs/pool) was used per gene to invalidate ($n = 8$). The median value is represented as a black line. The violin plot represents the full range of values where the width of the colored region represents the probability density of sample values at that level. Statistical test used is Student's *t*-test.

D Bar plot representing tumorsphere-forming efficiency (SFE) in SUM159 following gene silencing combinations. Statistical test used is Student's *t*-test ($n = 4$). Results are represented as mean ± SD.

© 2019 The Authors

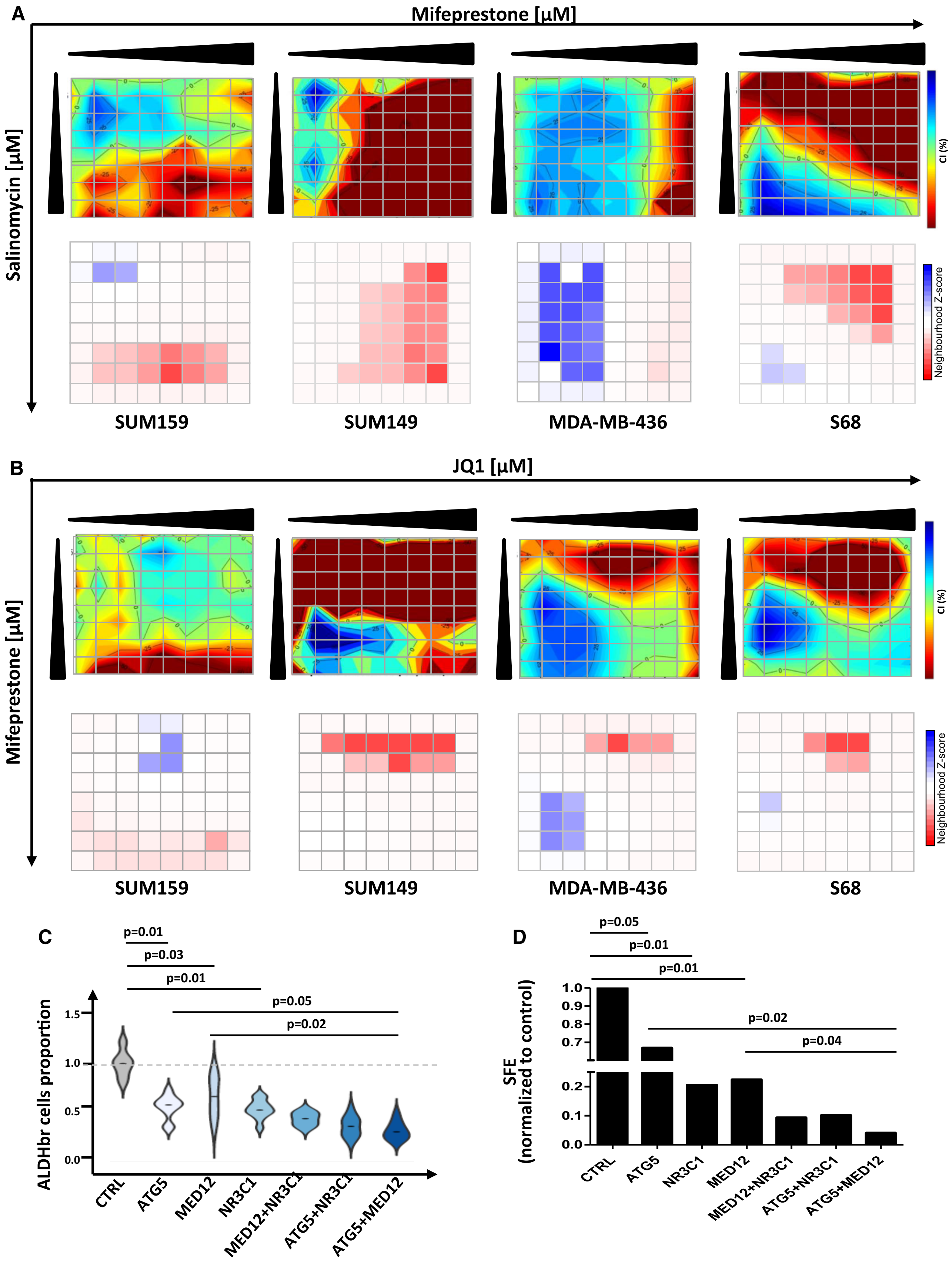

Figure EV1.

 © 2019 The Authors

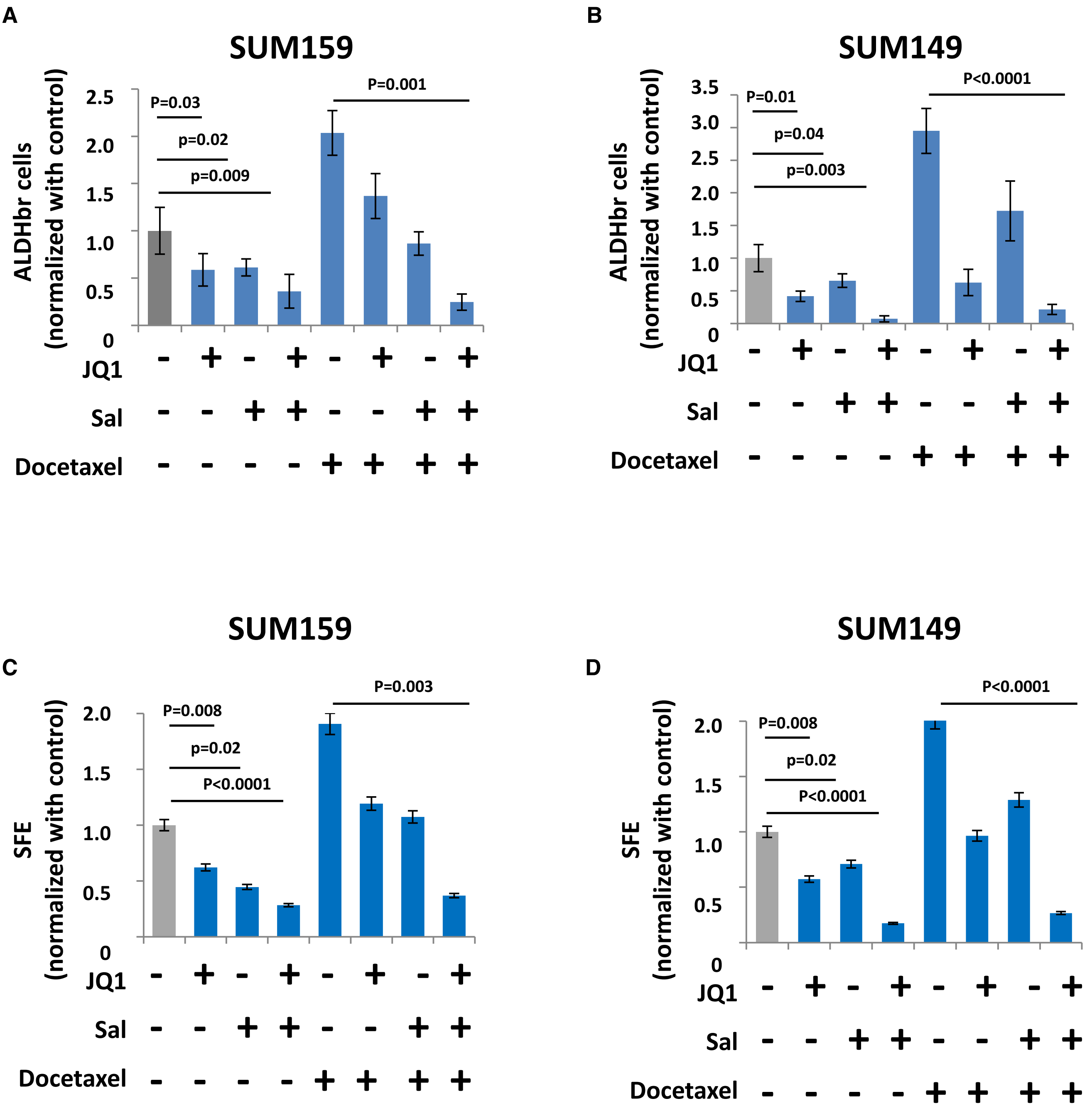

Figure EV2. Effect of the association of docetaxel with the salinomycin/JQ1 drug combination on the bCSC population.

A, B Representation of the proportion of residual ALDHbr cells following different treatment conditions in SUM159 and SUM149 cells. Statistical test used is Student's *t*-test ($n = 8$). Data represent mean ± SD.

C, D Primary tumorsphere-forming efficiency (SFE) of SUM159 and SUM149 cells under different treatment conditions. Statistical test used is pairwise chi-square test ($n = 4$). Data represent mean ± SD.

© 2019 The Authors

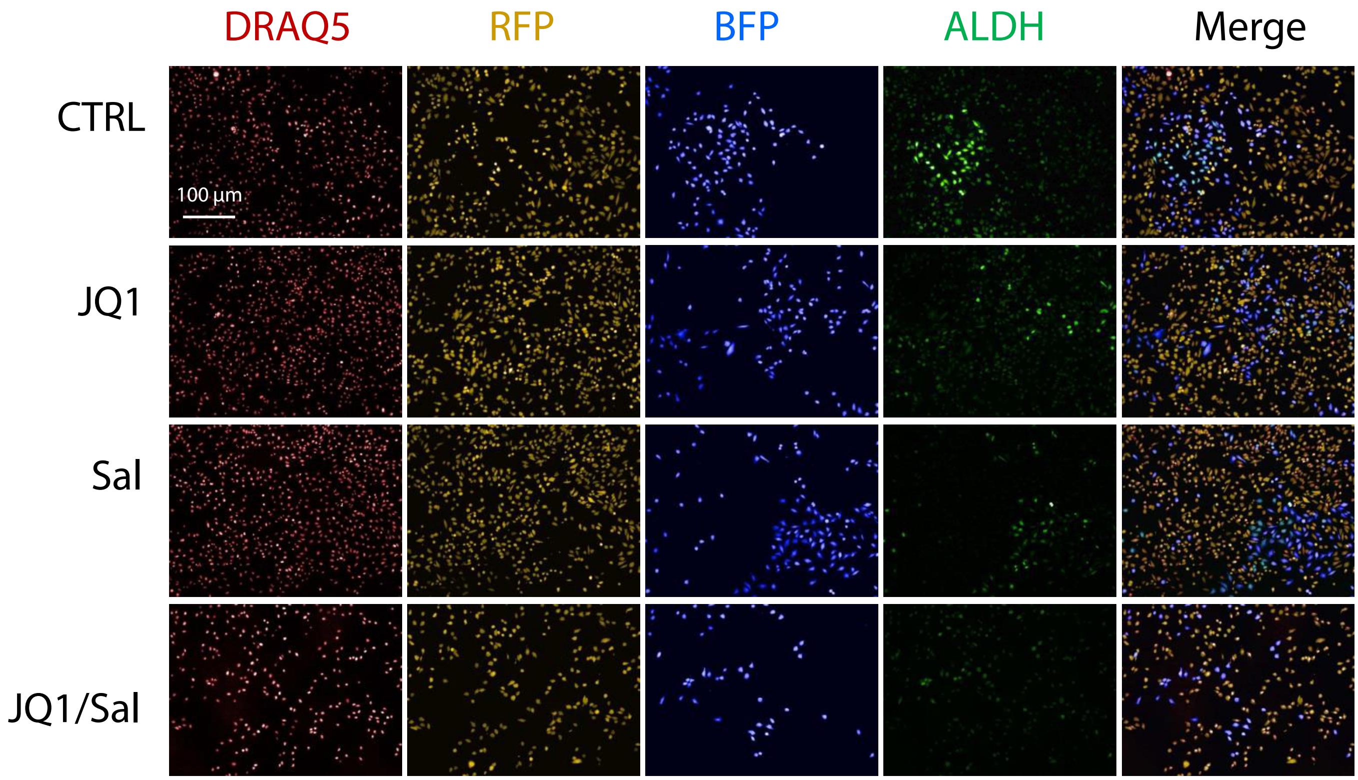

Figure EV3. Detection of the lineage tracing system.

Representative images of the high-content screening (HCS) captures. DRAQ5 nuclear DNA staining is shown in red, RFP^{+} cells are in orange, BFP^{+} cells are in blue, and ALDEFLUOR cellular staining is in green.

Source data are available online for this figure.

Figure EV4. *In vivo* limiting dilution assay and metastasis formation assay results.

- A Effect of JQ1 and salinomycin treatment on the tumor growth of CRCM404 (n = 10). The gray area corresponds to the treatment period. Data represent mean ± SD.
- B–D Reimplantation assay. Two-week treated PDXs were reimplanted, in serial dilutions, into new recipient mice, and tumor growth was monitored (CRCM404). Each curve represents the growth kinetic from one individual injection.
- E Effect of JQ1 and salinomycin treatment on the tumor growth of CRCM 494 (n = 10). The gray area corresponds to the treatment period. Data represent mean ± SD.
- F–H Reimplantation assay. Two-week treated PDXs were reimplanted, in serial dilutions, into new recipient mice, and tumor growth was monitored (CRCM494). Each curve represents the growth kinetic from one individual injection.
- I Table showing the number of outgrowths generated in NSG mouse fat pads as a function of the amount of injected cells isolated from CRCM404, CRCM434, and CRCM494 PDXs following JQ1, salinomycin, JQ1/salinomycin combination, or placebo treatment.
- J Effect of JQ1 and salinomycin treatments on the metastasis formation of CRCM404. Metastasis formation was monitored using bioluminescence imaging in mice livers and lungs.

© 2019 The Authors

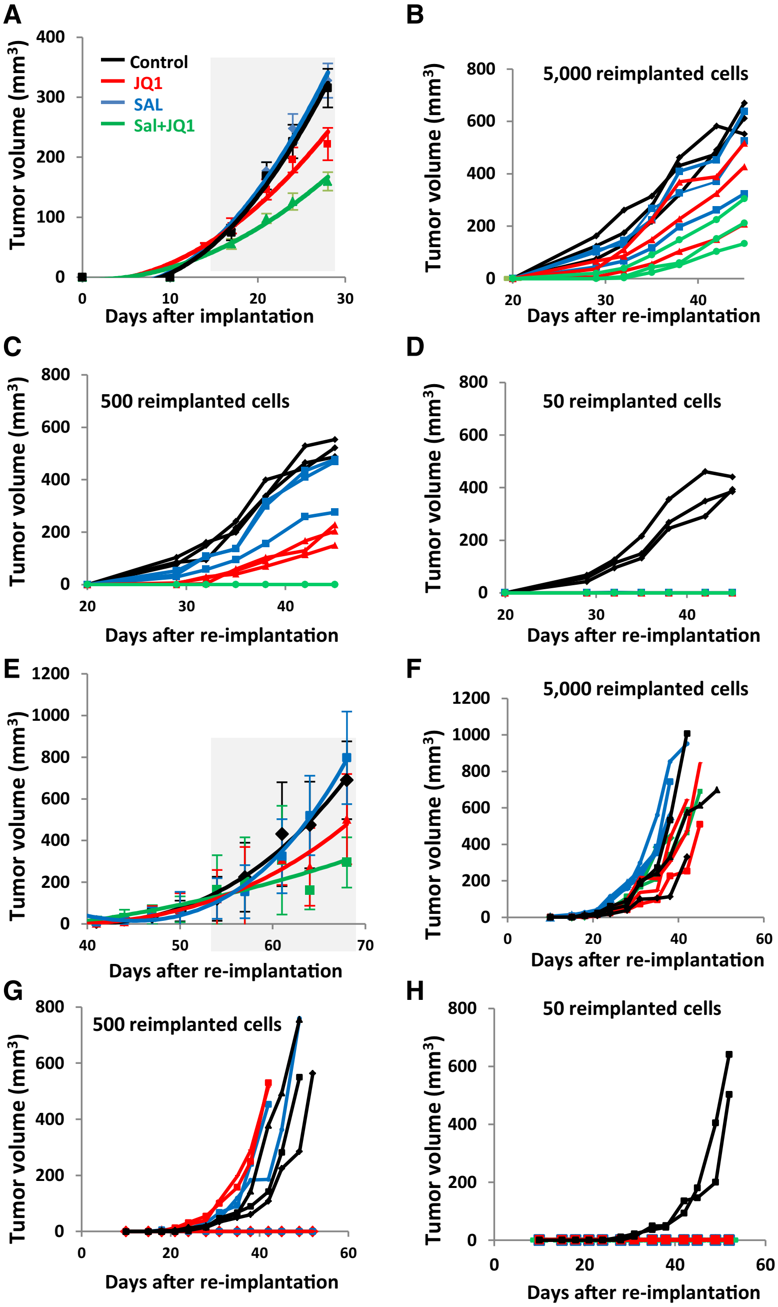

I

	Limiting Dilution Assay (number of injected cells) 10	50	500	5000
CRCM434 CTRL (outgrowth/injected fatpads)	0/1	4/4	3/3	3/3
CRCM434 JQ1 (outgrowth/injected fatpads)		0/4	2/3	3/3
CRCM434 Salinomycin (outgrowth/injected fatpads)		0/4	3/3	3/3
CRCM434 JQ1 + Sal (outgrowth/injected fatpads)		0/4	0/3	3/3
CRCM404 CTRL (outgrowth/injected fatpads)		3/4	3/3	3/3
CRCM404 JQ1 (outgrowth/injected fatpads)		0/4	3/3	3/3
CRCM404 Salinomycin (outgrowth/injected fatpads)		0/4	3/3	3/3
CRCM404 JQ1 + Sal (outgrowth/injected fatpads)		0/4	0/3	3/3
CRCM494 CTRL (outgrowth/injected fatpads)		2/4	3/3	3/3
CRCM494 JQ1 (outgrowth/injected fatpads)		0/4	2/3	3/3
CRCM494 Salinomycin (outgrowth/injected fatpads)		0/4	2/3	3/3
CRCM494 JQ1 + Sal (outgrowth/injected fatpads)		0/4	0/3	2/3

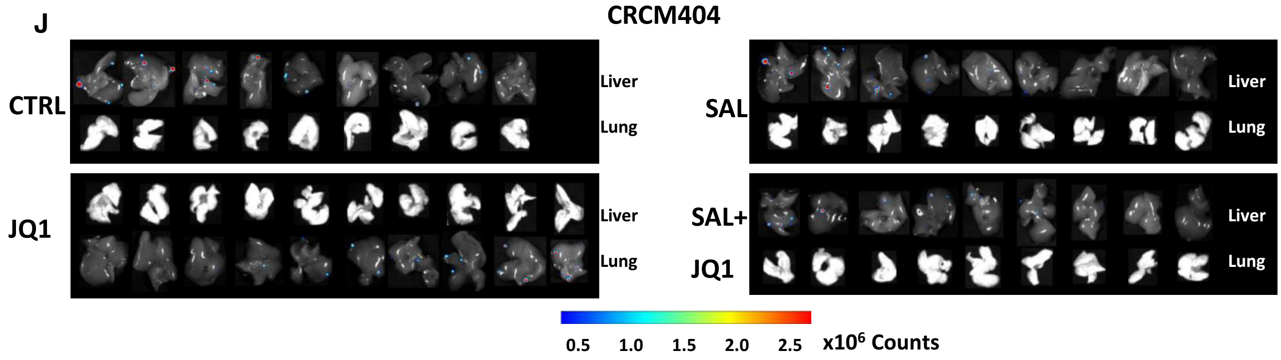

Figure EV4.

Figure EV5. Effect of salinomycin/JQ1 treatment on cell proliferation, apoptosis, and subnetwork gene expression in PDXs.

A Representative KI67 immunostaining (brown staining) in different treated tumors (right panel). Counterstaining is in blue. Quantification of the proportion of proliferative $KI67^+$ cells (left panel). Statistical test used is *t*-test. Data represent mean ± SD (n = 3).

B Representative cleaved caspase-3 immunostaining (brown staining) in different treated tumors (right panel). Counterstaining is in blue. Quantification of the proportion of apoptotic, cleaved caspase-3^+ cells (left panel). Statistical test used is *t*-test. Data represent mean ± SD (n = 3).

C Table showing the detailed results of the logistic regression analysis for each subnetwork.

© 2019 The Authors

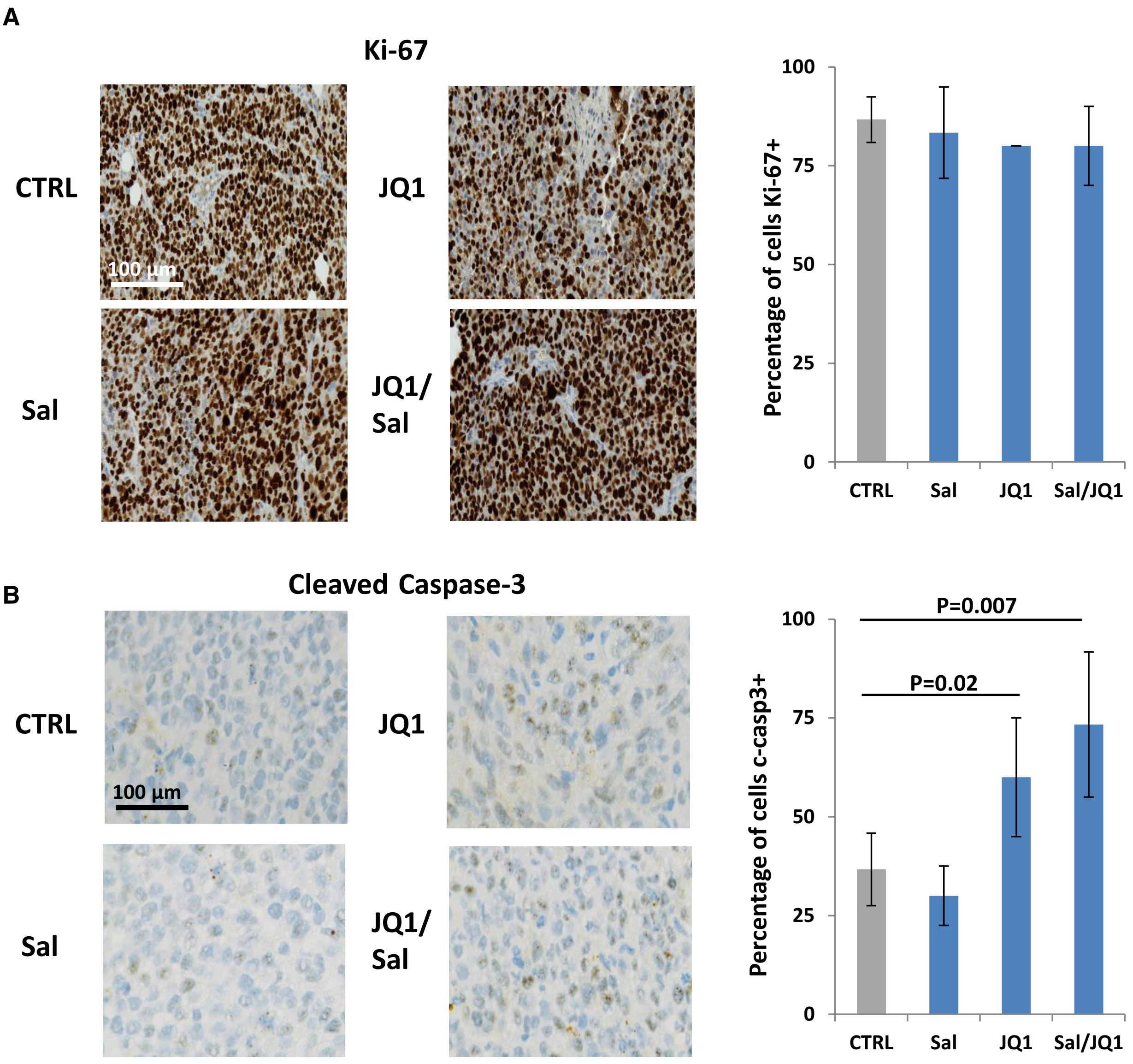

C

	Comb vs. Ctrl, logistic regression			
	N	Odds-ratio [95%CI]	p-value	FDR
Subnet-1 (KPNA2-Related network)	6	0.87 [0.82-0.93]	2.71E-02	0.05
**Subnet-2 (TNF signaling)**	6	1.18 [1.14-1.22]	1.09E-03	4.00E-03
**Subnet-5 (EP300-related network)**	6	1.04 [1.03-1.06]	4.20E-03	1.15E-02
Subnet-3 (Proteasome)	6	0.84 [0.66-1.07]	0.305	0.34
Subnet-4 (Spliceosome)	6	1.06 [0.98-1.16]	0.285	0.34
Sunbet-7 (Nuclear Receptors network)	6	1.11 [1.04-1.18]	0.053	0.08
**Subnet-6 (Mediator Complex)**	6	0.90 [0.89-0.92]	6.66E-04	3.66E-03
**Subnet-10 (ADARB1-related network)**	6	1.12 [1.11-1.13]	1.95E-05	2.15E-04
Subnet-8	6	0.98 [0.91-1.06]	0.692	0.69
**Subnet-9 (Autophagy)**	6	0.97 [0.96-0.98]	1.30E-02	2.86E-02
Subnet-11 (Ribosomal_Machinery)	6	0.89 [0.82-0.97]	0.099	0.14

Figure EV5.
