# Supplementary figures and images for: A genome‐wide RNAi screen reveals essential therapeutic targets of breast cancer stem cells
Source: EMBO Mol Med. 2019 Sep 2;11(10):e9930. doi: 10.15252/emmm.201809930 (PMC6783652; doi:10.15252/emmm.201809930)

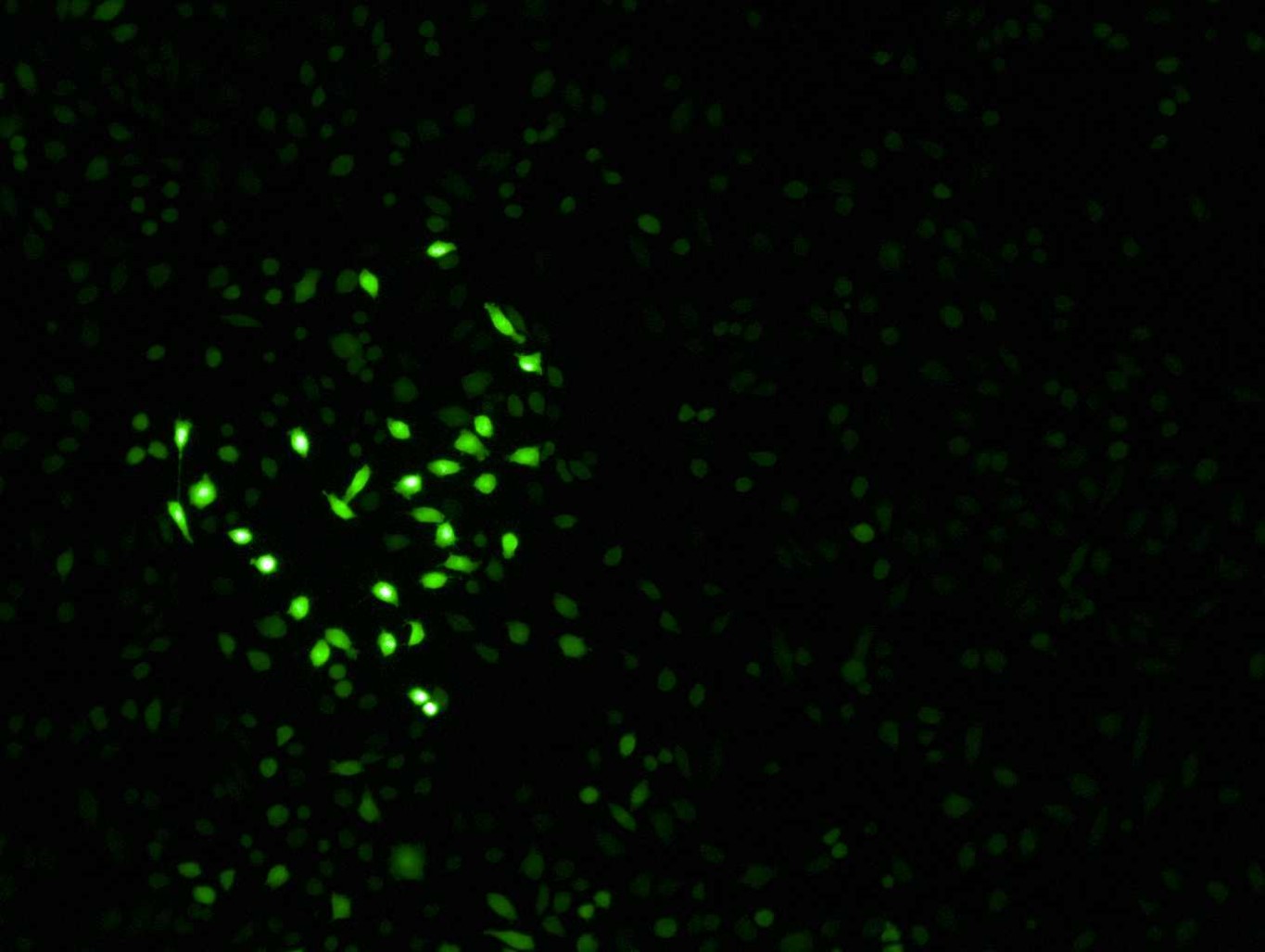

Supplement: Supplementary file 6 — Source Data for Expanded View [file EMMM-11-e9930-s008.zip › Fig_EV3/CTRL_ALDH.jpg]

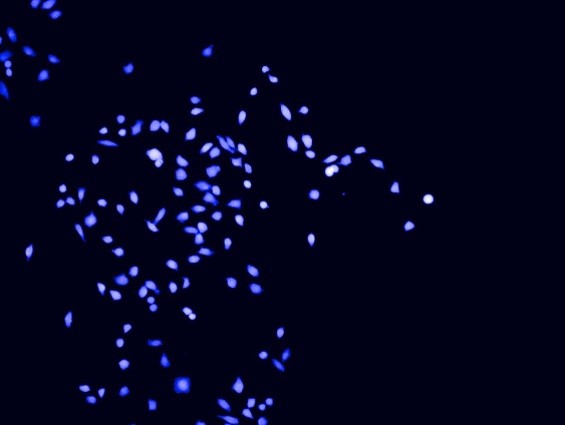

Supplement: Supplementary file 6 — Source Data for Expanded View [file EMMM-11-e9930-s008.zip › Fig_EV3/CTRL_BFP.jpg]

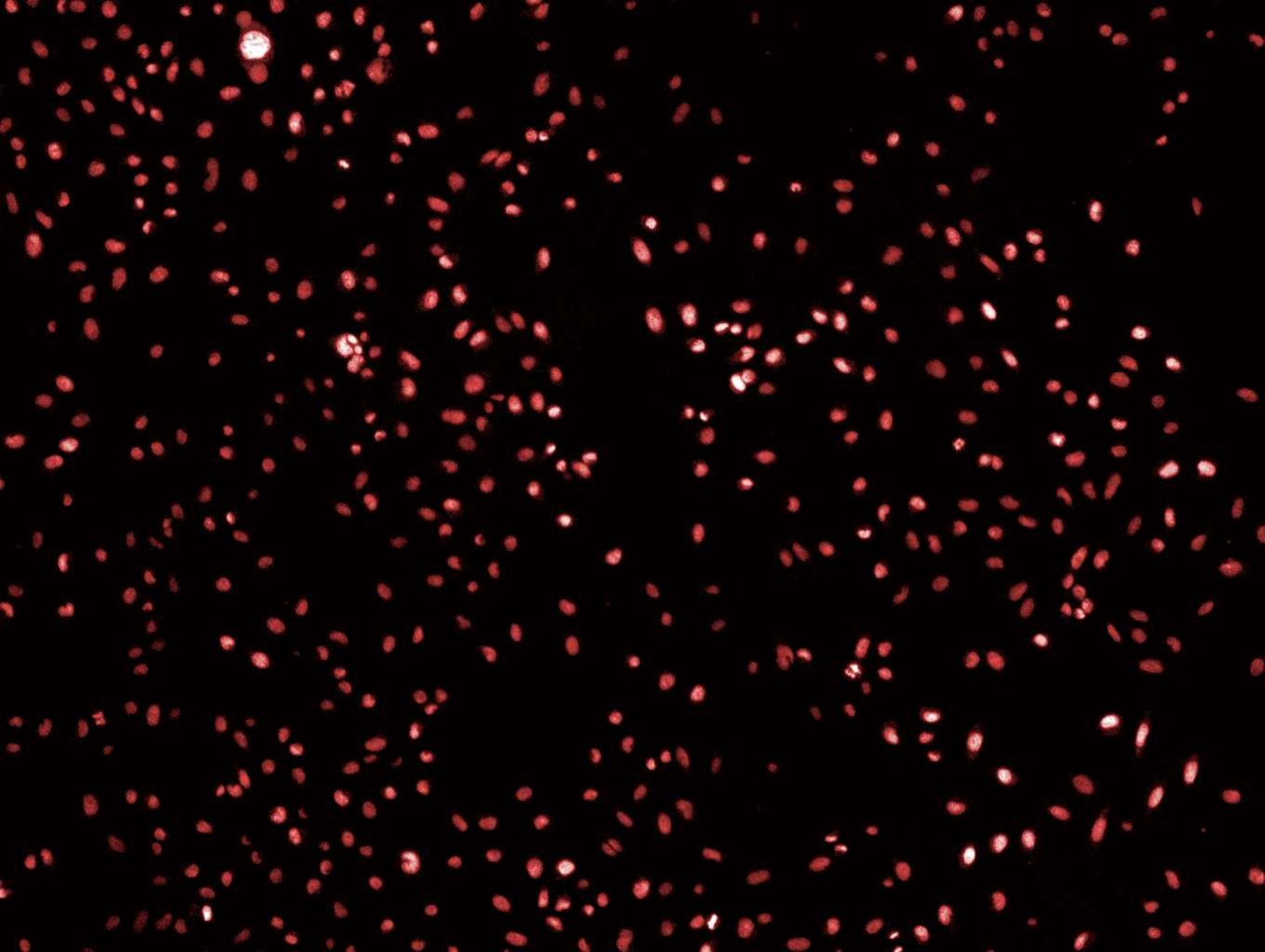

Supplement: Supplementary file 6 — Source Data for Expanded View [file EMMM-11-e9930-s008.zip › Fig_EV3/CTRL_DRAQ5.jpg]

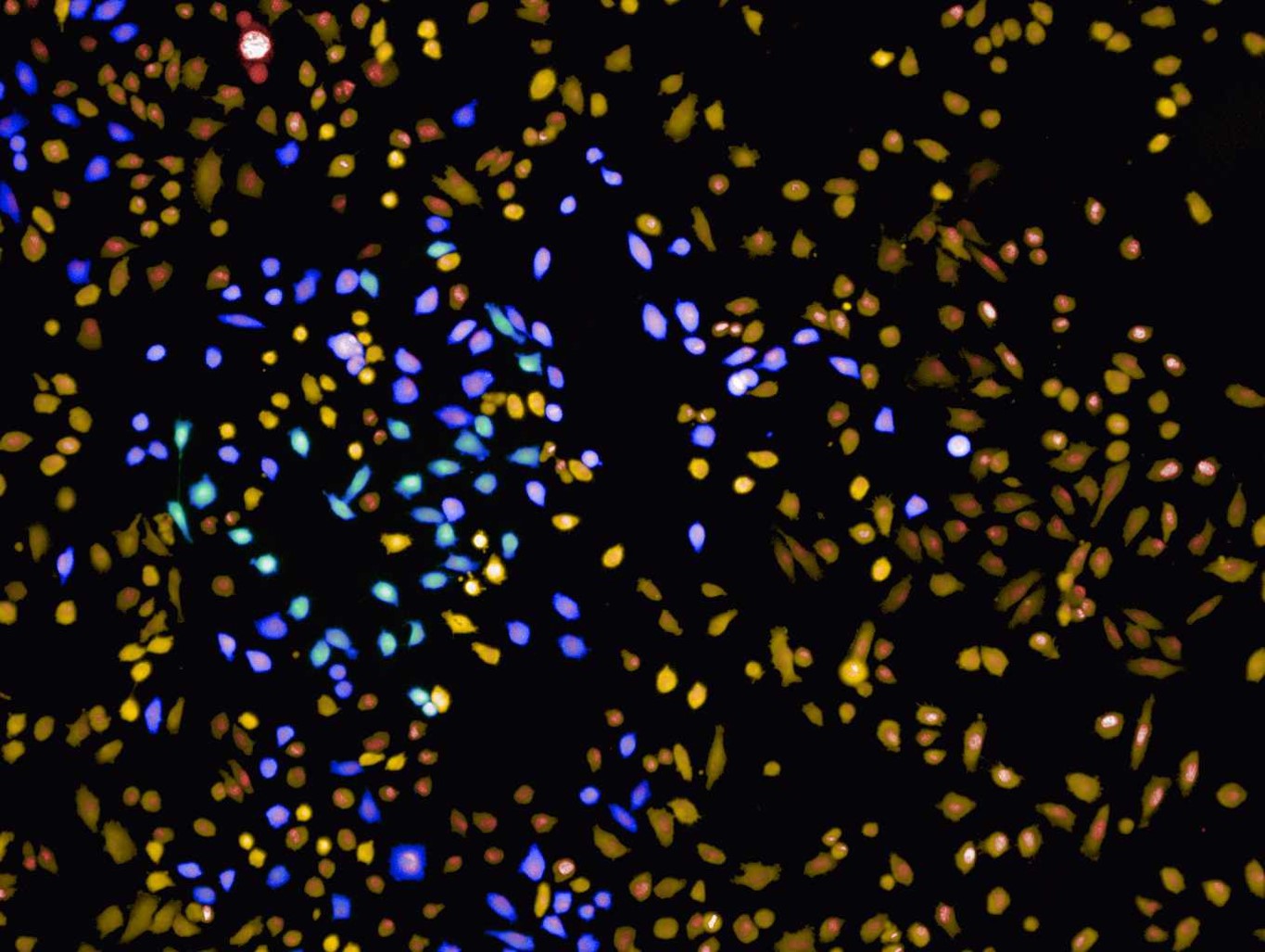

Supplement: Supplementary file 6 — Source Data for Expanded View [file EMMM-11-e9930-s008.zip › Fig_EV3/CTRL_Merge.jpg]

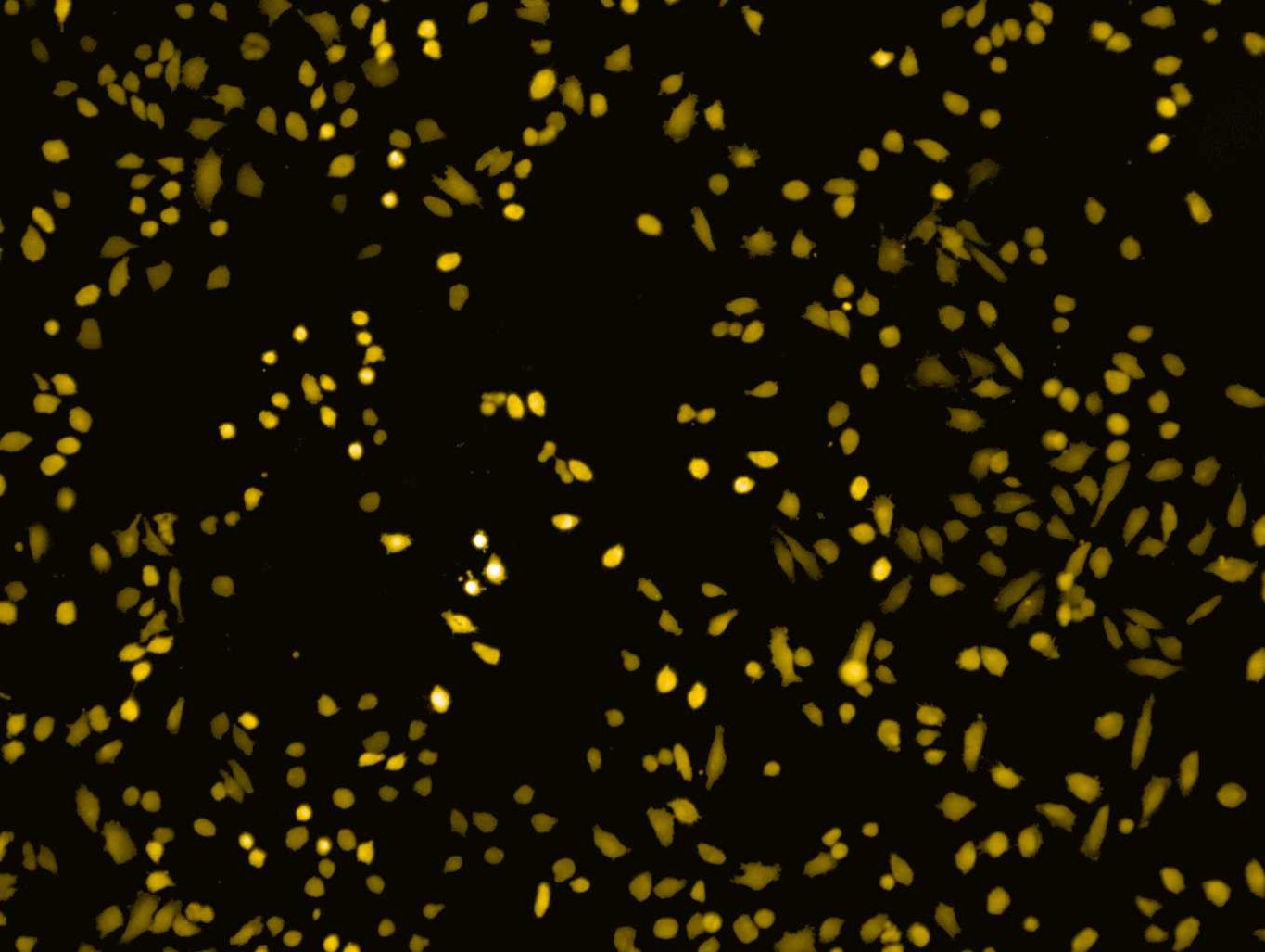

Supplement: Supplementary file 6 — Source Data for Expanded View [file EMMM-11-e9930-s008.zip › Fig_EV3/CTRL_RFP.jpg]

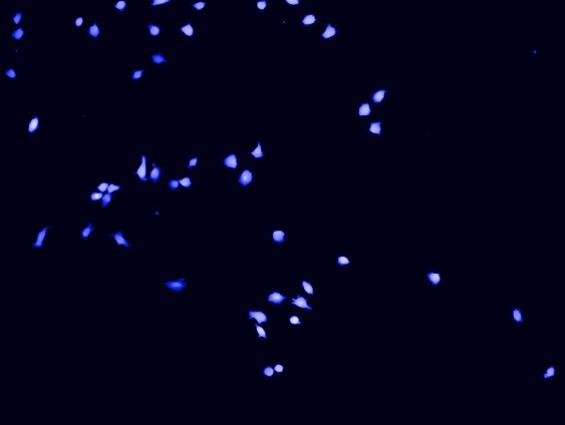

Supplement: Supplementary file 6 — Source Data for Expanded View [file EMMM-11-e9930-s008.zip › Fig_EV3/JQ1-SAL_BFP.jpg]

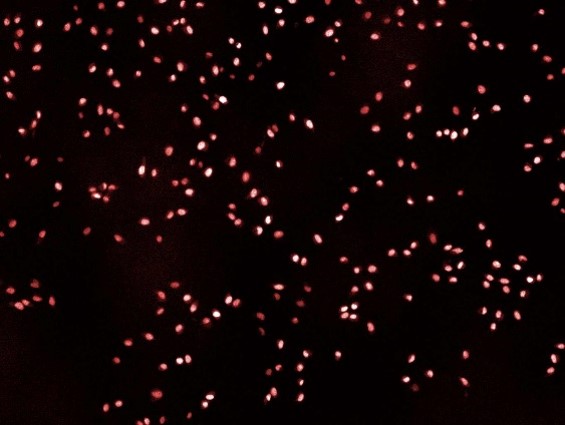

Supplement: Supplementary file 6 — Source Data for Expanded View [file EMMM-11-e9930-s008.zip › Fig_EV3/JQ1-SAL_DRAQ5.jpg]

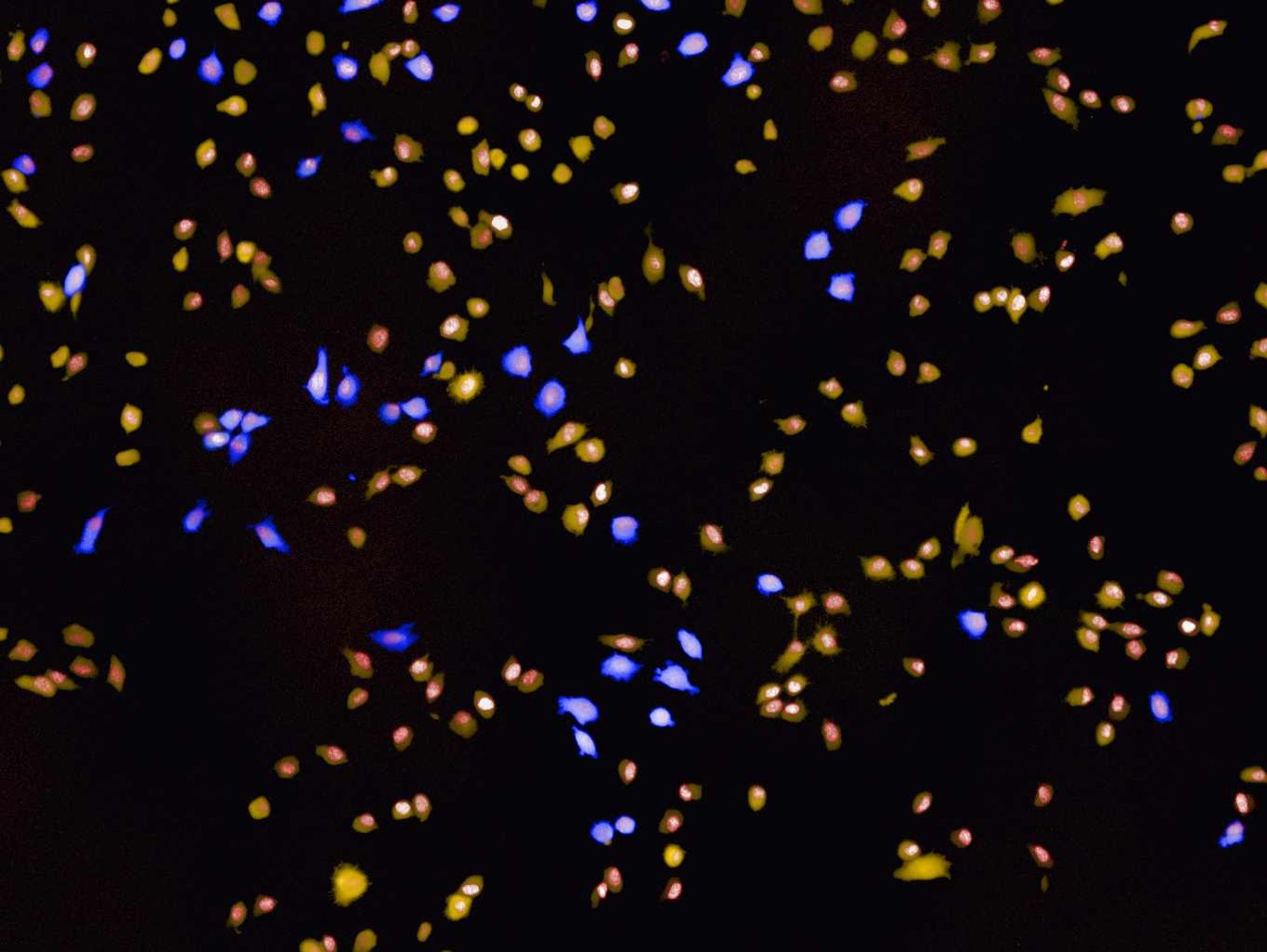

Supplement: Supplementary file 6 — Source Data for Expanded View [file EMMM-11-e9930-s008.zip › Fig_EV3/JQ1-SAL_Merge.jpg]

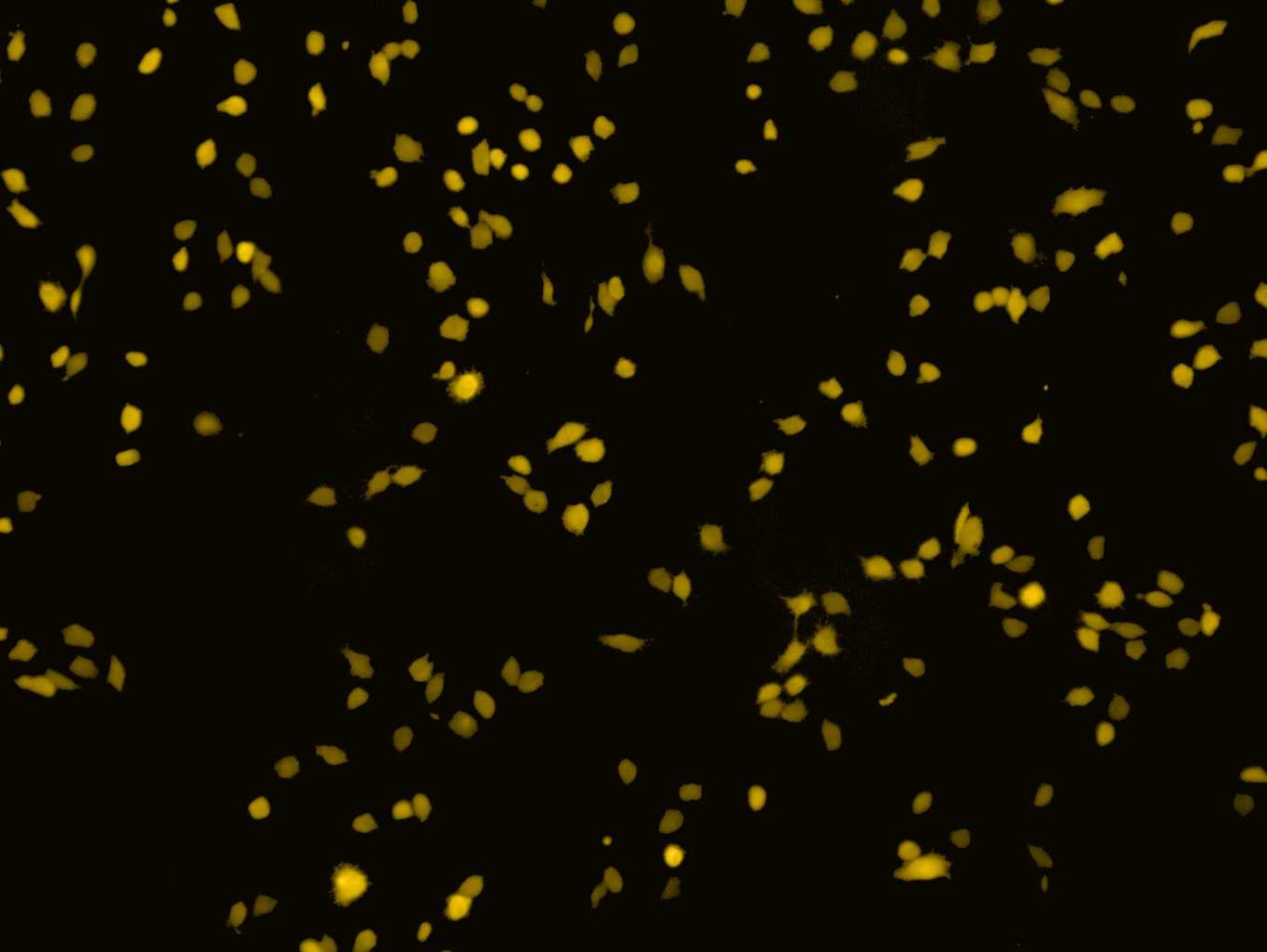

Supplement: Supplementary file 6 — Source Data for Expanded View [file EMMM-11-e9930-s008.zip › Fig_EV3/JQ1-SAL_RFP.jpg]

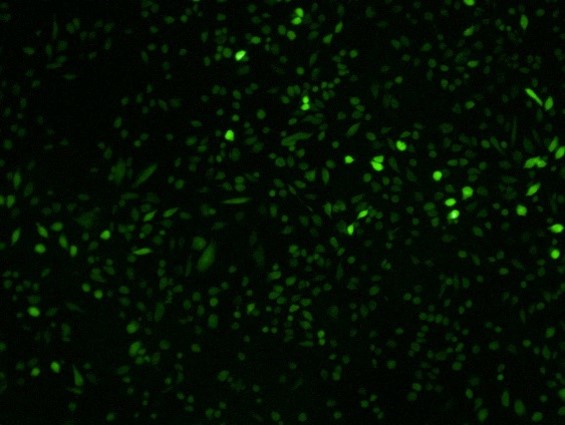

Supplement: Supplementary file 6 — Source Data for Expanded View [file EMMM-11-e9930-s008.zip › Fig_EV3/JQ1_ALDH.jpg]

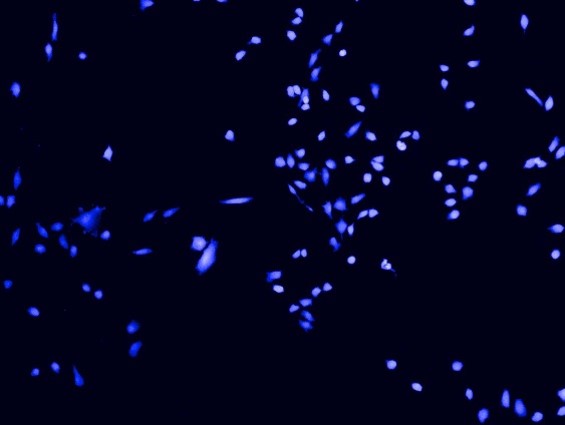

Supplement: Supplementary file 6 — Source Data for Expanded View [file EMMM-11-e9930-s008.zip › Fig_EV3/JQ1_BFP.jpg]

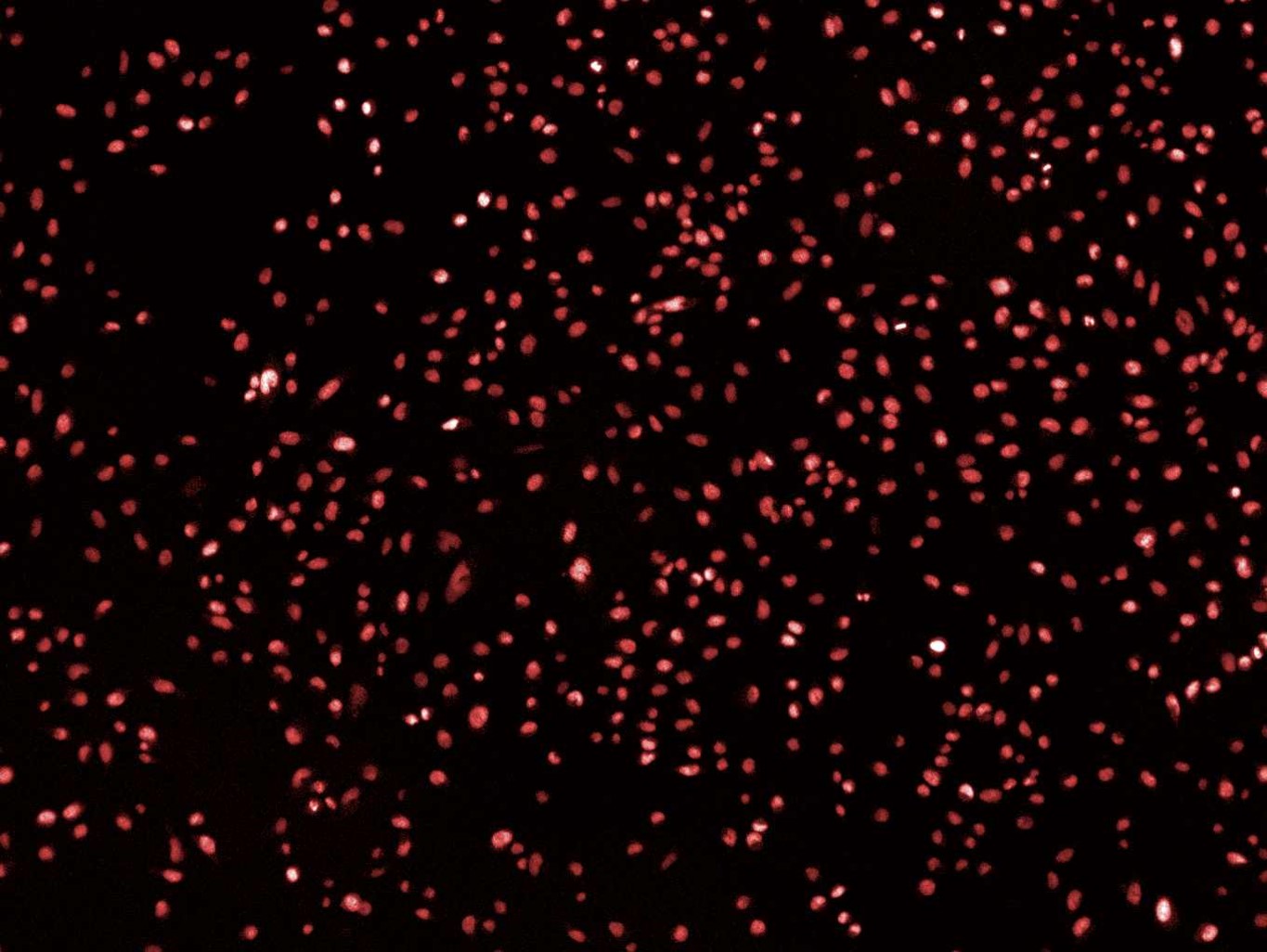

Supplement: Supplementary file 6 — Source Data for Expanded View [file EMMM-11-e9930-s008.zip › Fig_EV3/JQ1_DRAQ5.jpg]

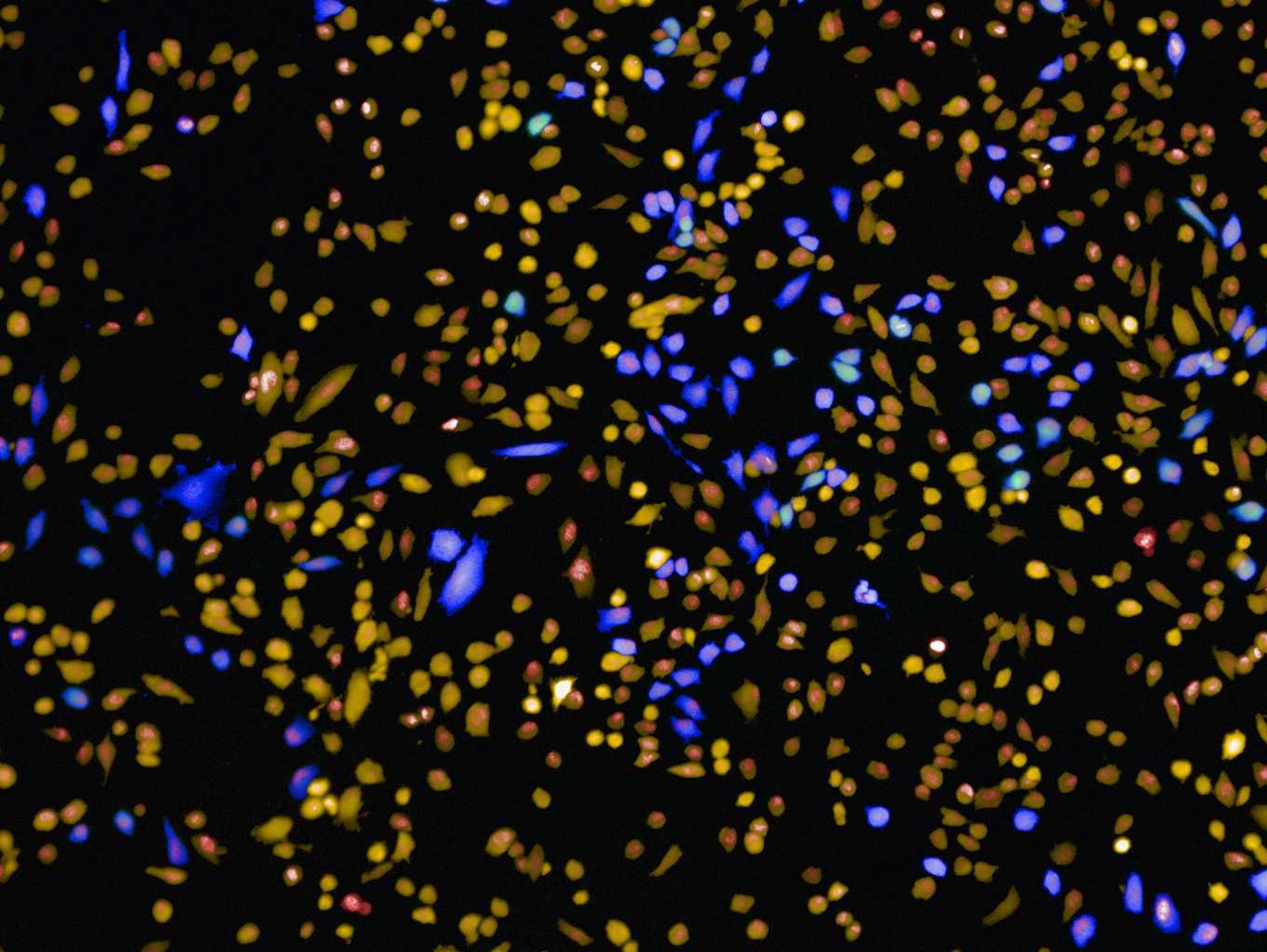

Supplement: Supplementary file 6 — Source Data for Expanded View [file EMMM-11-e9930-s008.zip › Fig_EV3/JQ1_Merge.jpg]

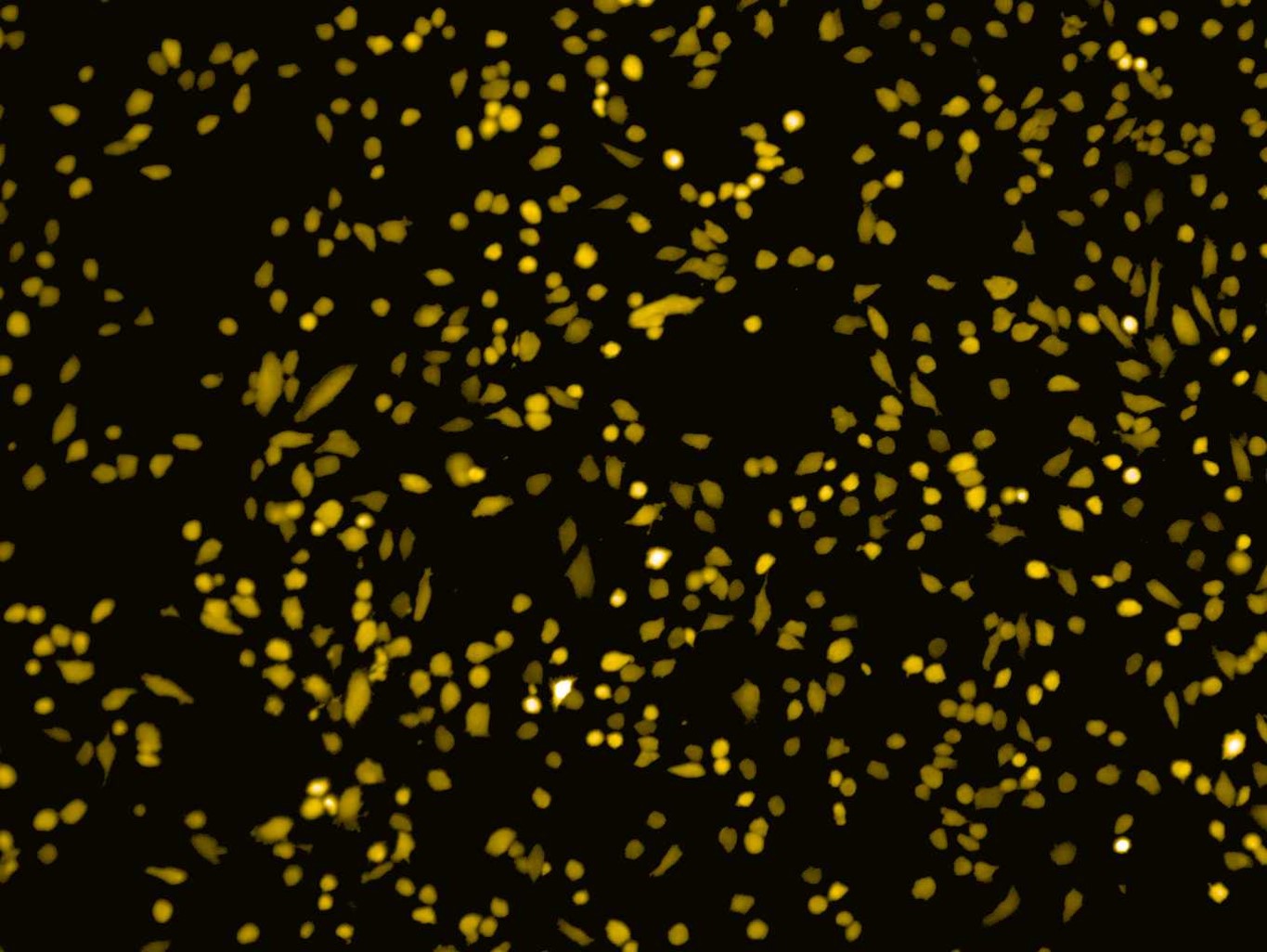

Supplement: Supplementary file 6 — Source Data for Expanded View [file EMMM-11-e9930-s008.zip › Fig_EV3/JQ1_RFP.jpg]

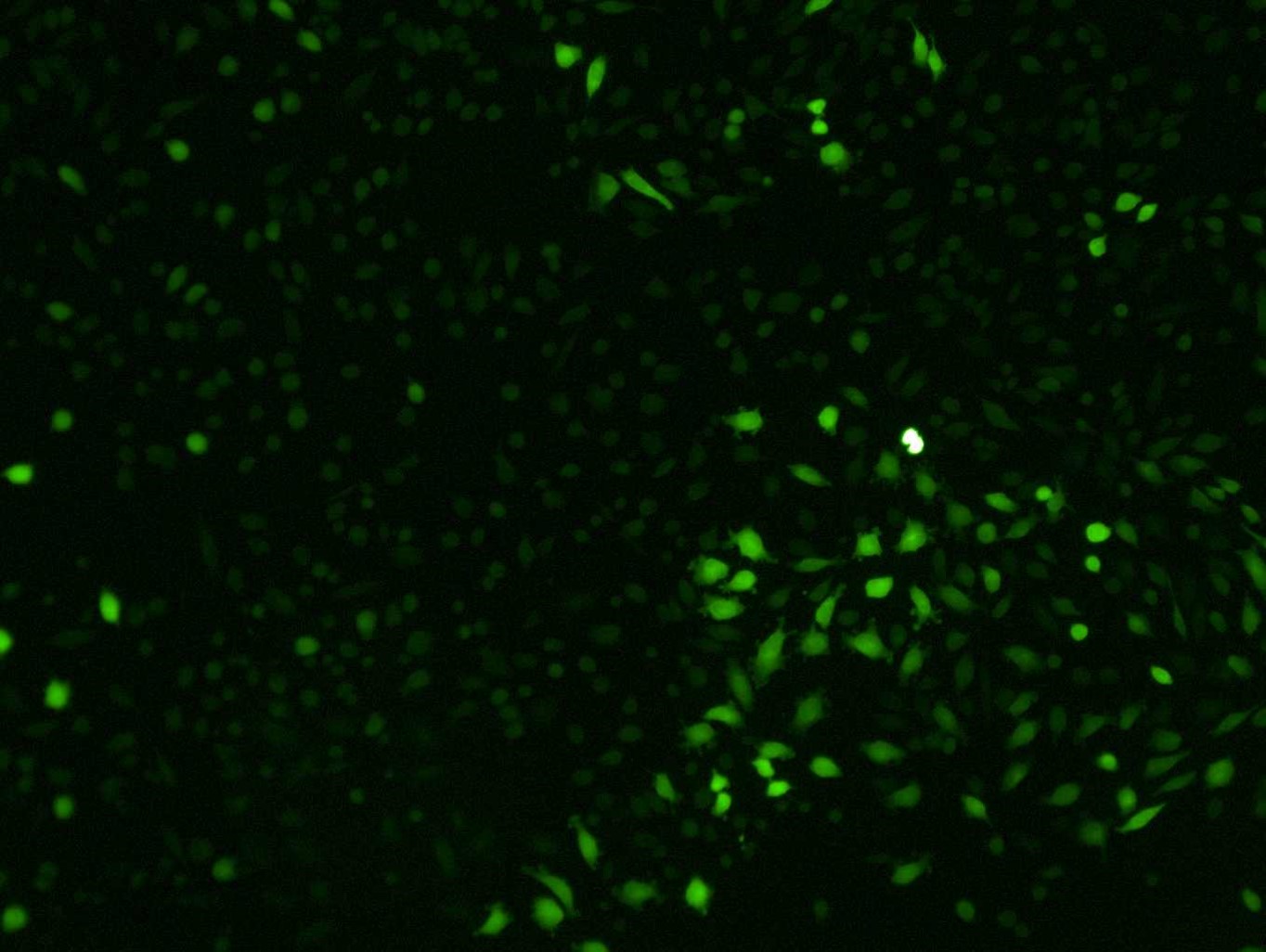

Supplement: Supplementary file 6 — Source Data for Expanded View [file EMMM-11-e9930-s008.zip › Fig_EV3/SAL_ALDH.jpg]

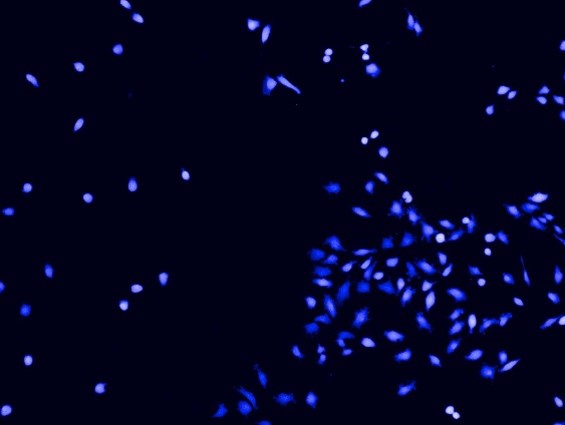

Supplement: Supplementary file 6 — Source Data for Expanded View [file EMMM-11-e9930-s008.zip › Fig_EV3/SAL_BFP.jpg]

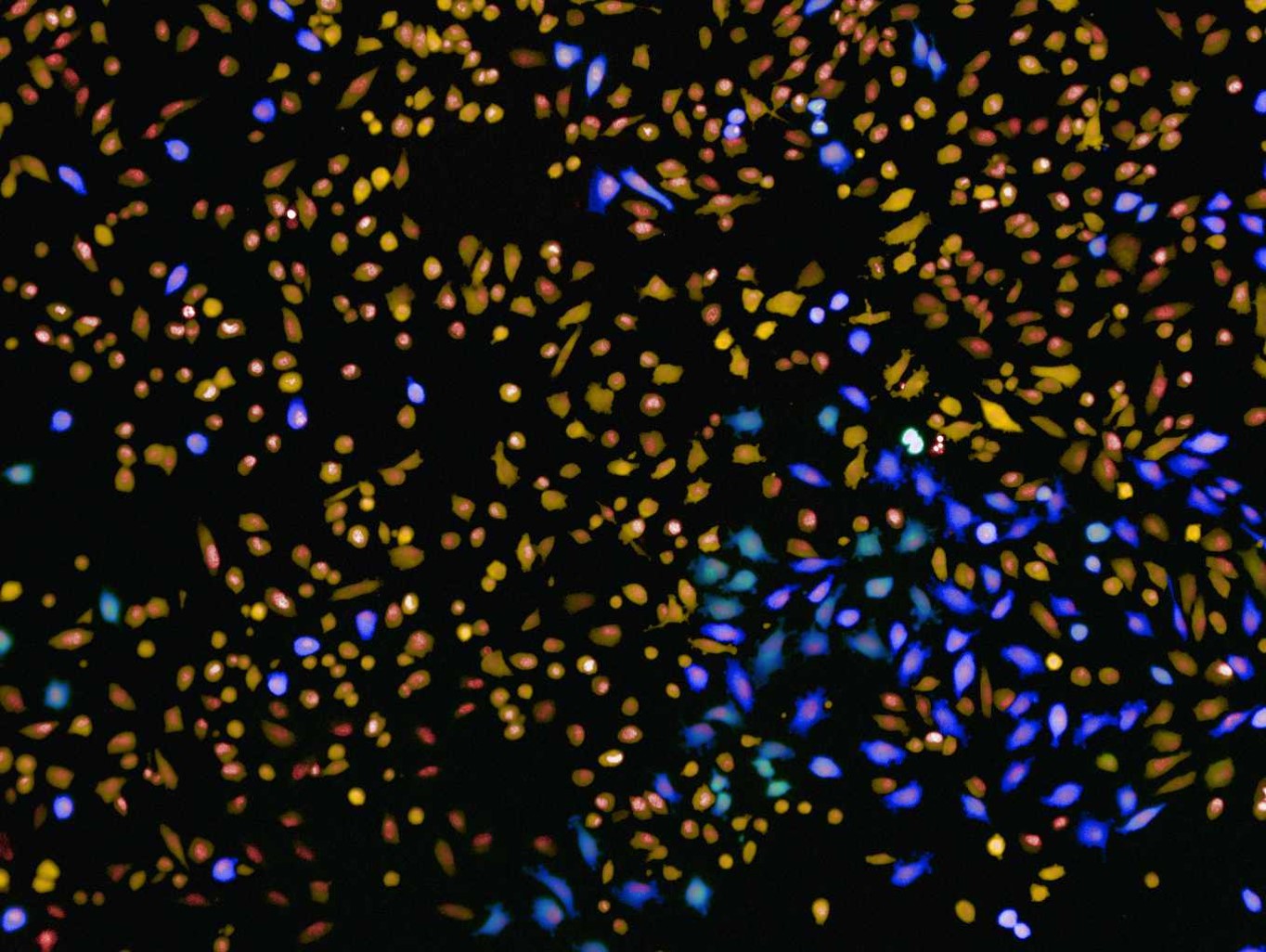

Supplement: Supplementary file 6 — Source Data for Expanded View [file EMMM-11-e9930-s008.zip › Fig_EV3/SAL_Merge.jpg]

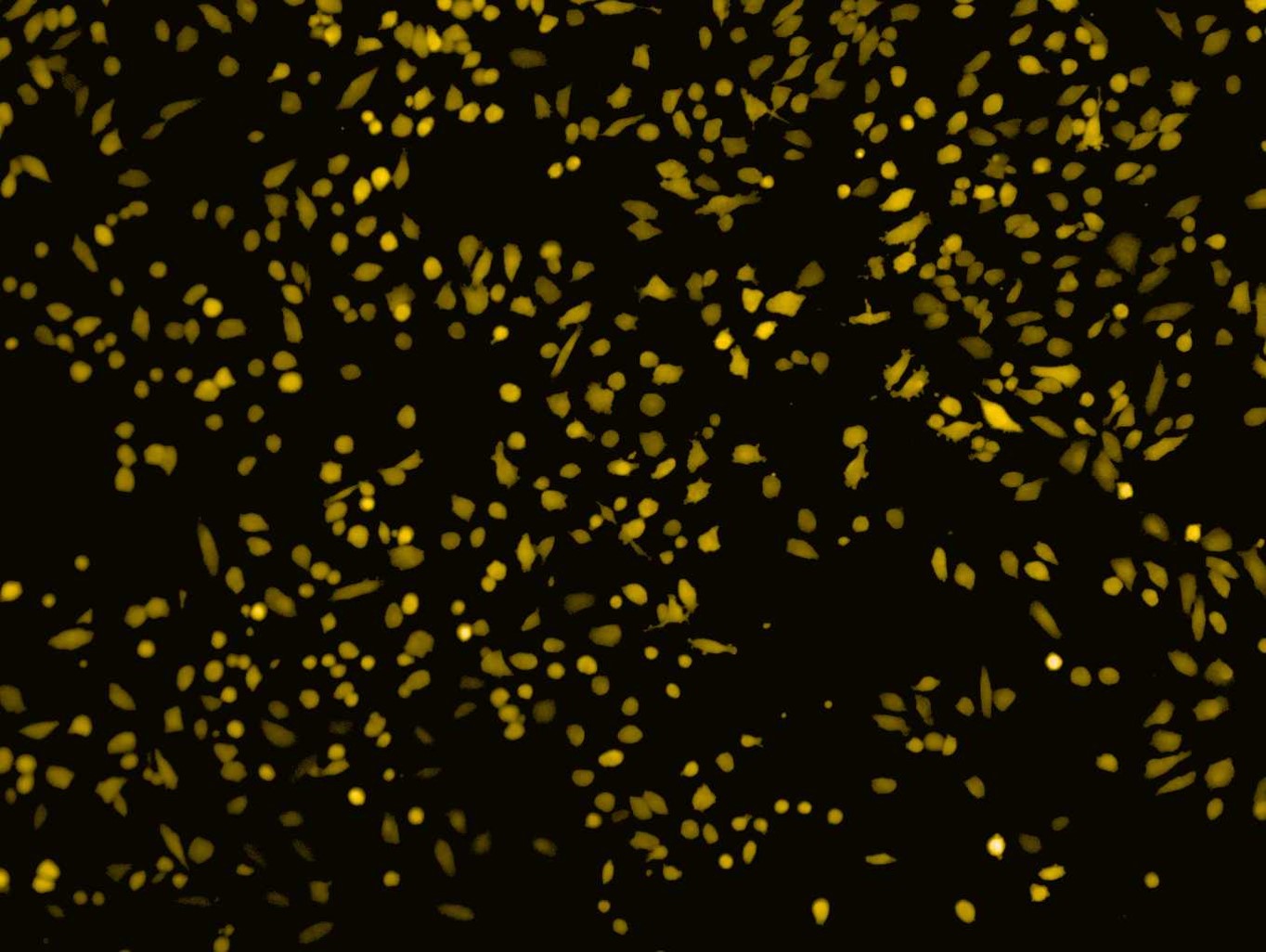

Supplement: Supplementary file 6 — Source Data for Expanded View [file EMMM-11-e9930-s008.zip › Fig_EV3/SAL_RFP.jpg]

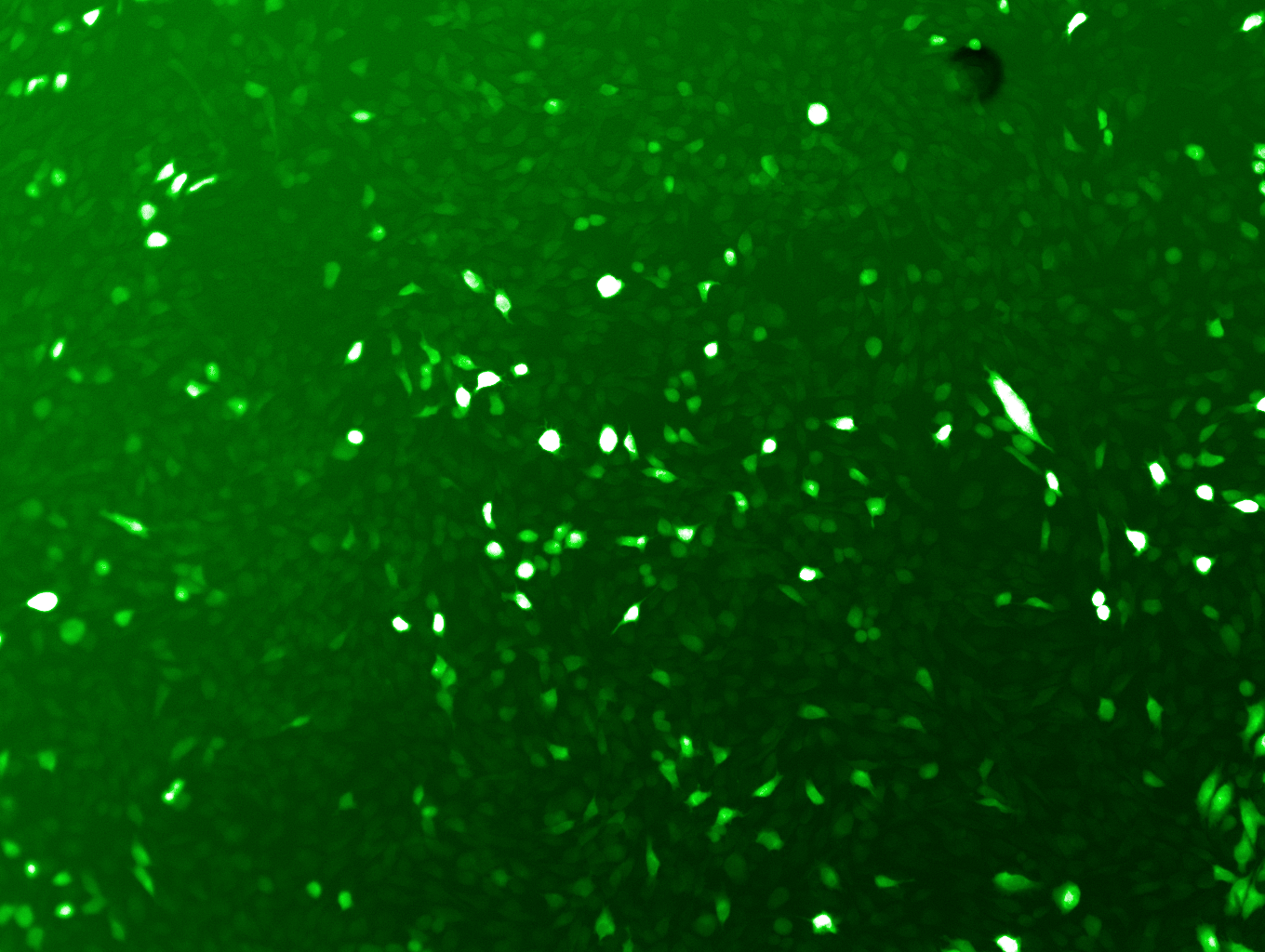

Supplement: Supplementary file 8 — Source Data for Figure 1 [file EMMM-11-e9930-s006.zip › Fig_1/Fig1B_ALDEFLUOR.png]

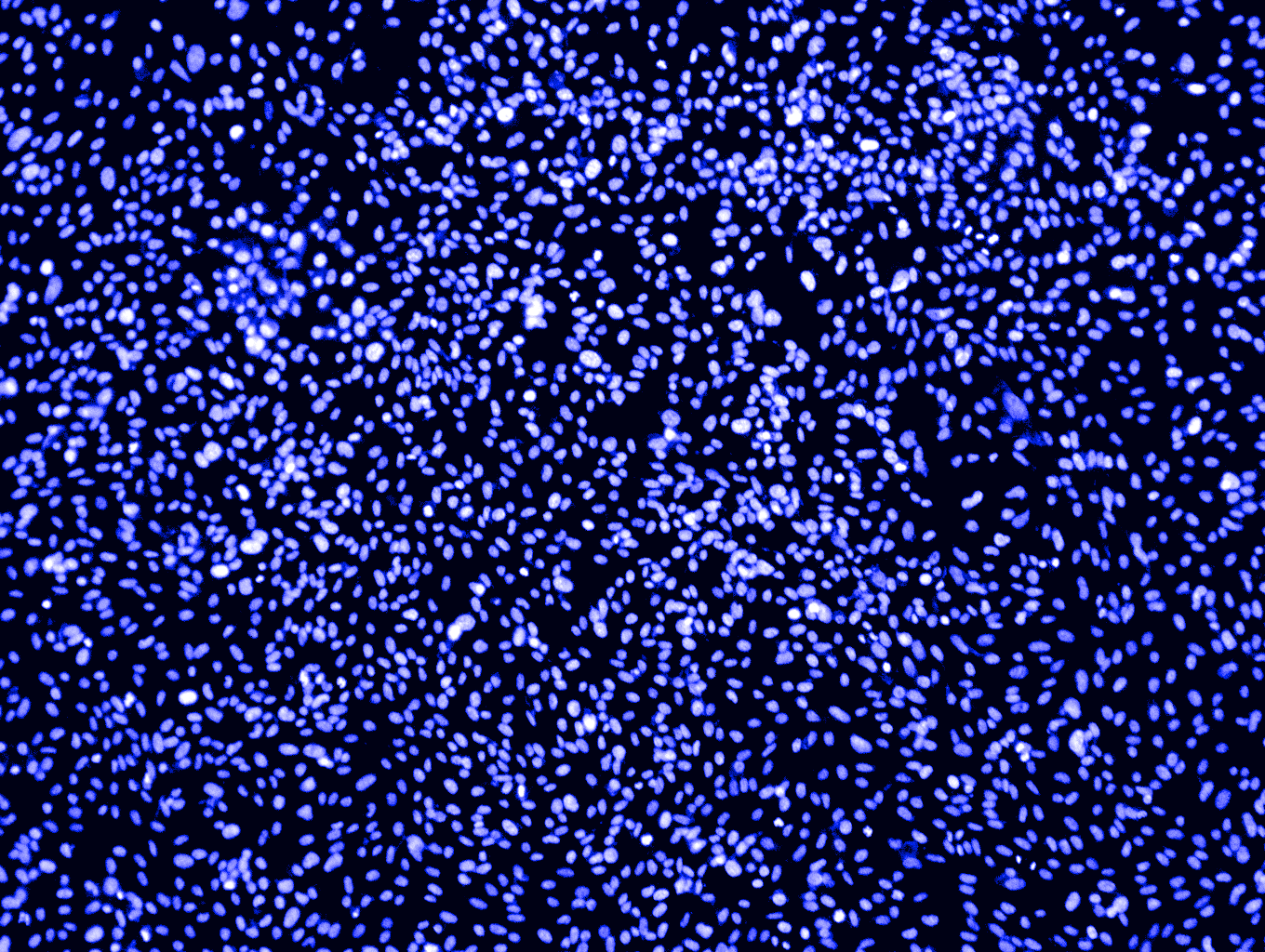

Supplement: Supplementary file 8 — Source Data for Figure 1 [file EMMM-11-e9930-s006.zip › Fig_1/Fig1B_Hoechst.png]

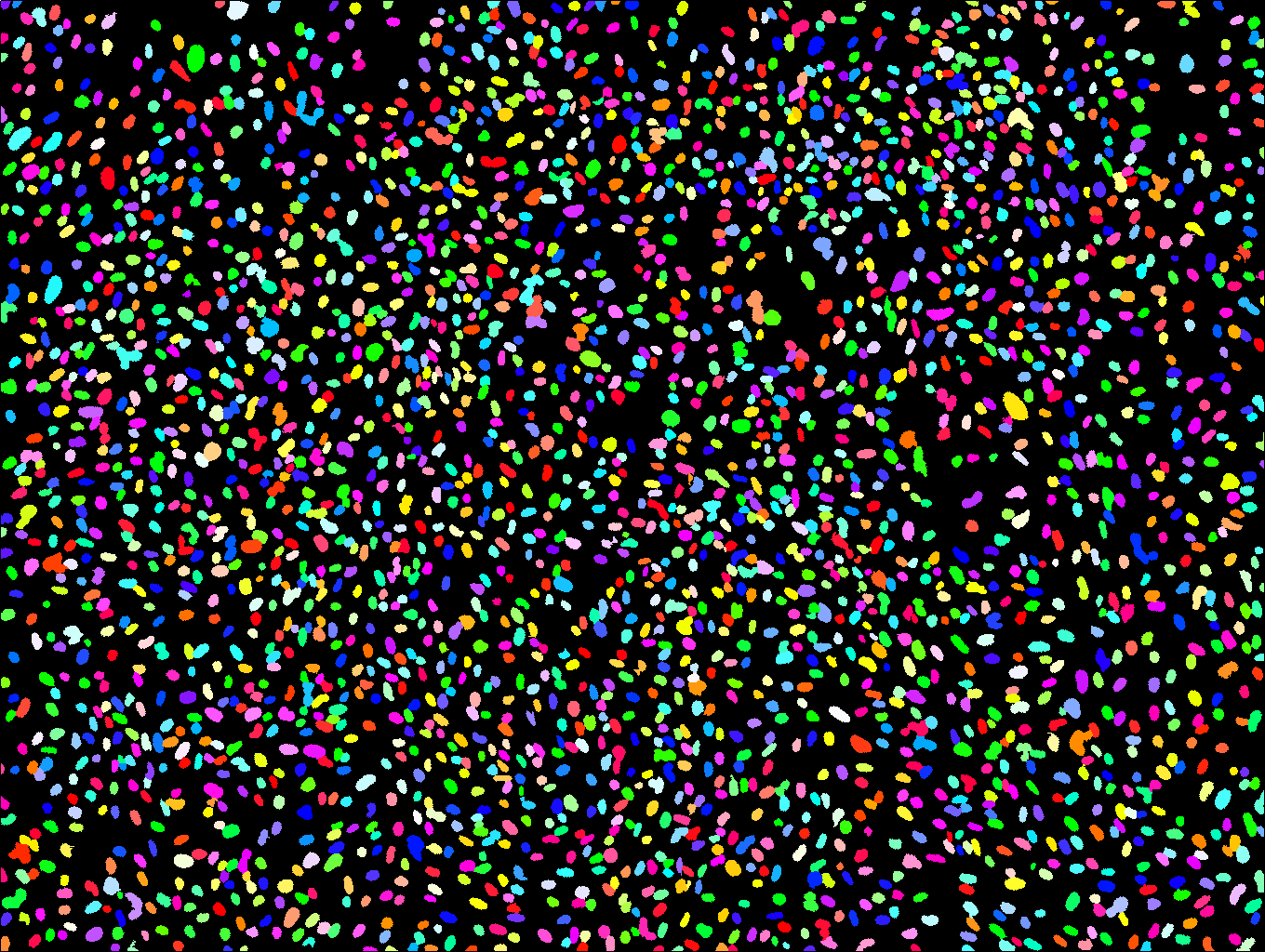

Supplement: Supplementary file 8 — Source Data for Figure 1 [file EMMM-11-e9930-s006.zip › Fig_1/Fig1B_Nuclear_ROI.png]

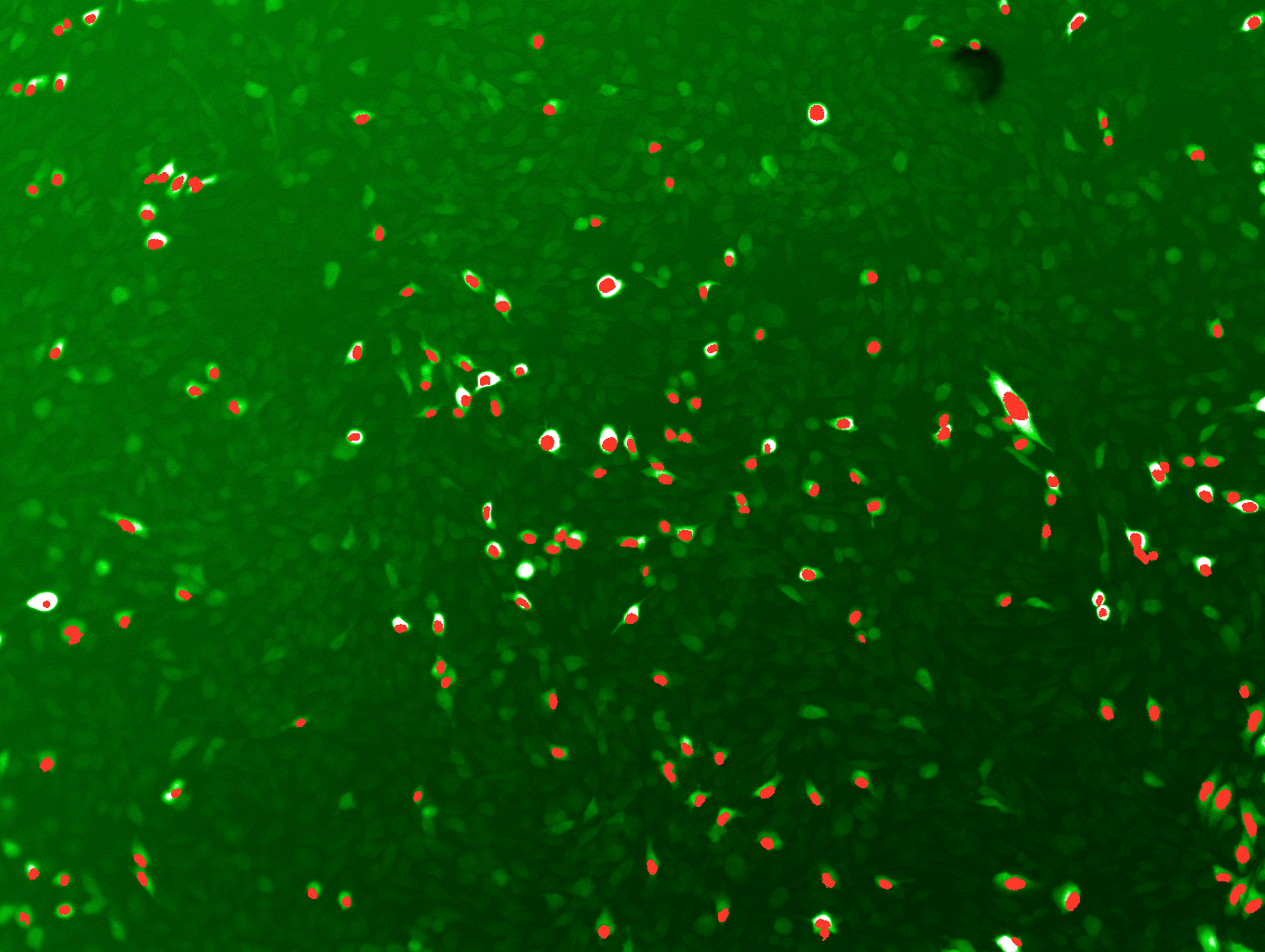

Supplement: Supplementary file 8 — Source Data for Figure 1 [file EMMM-11-e9930-s006.zip › Fig_1/Fig1B_Selected_CSC.png]

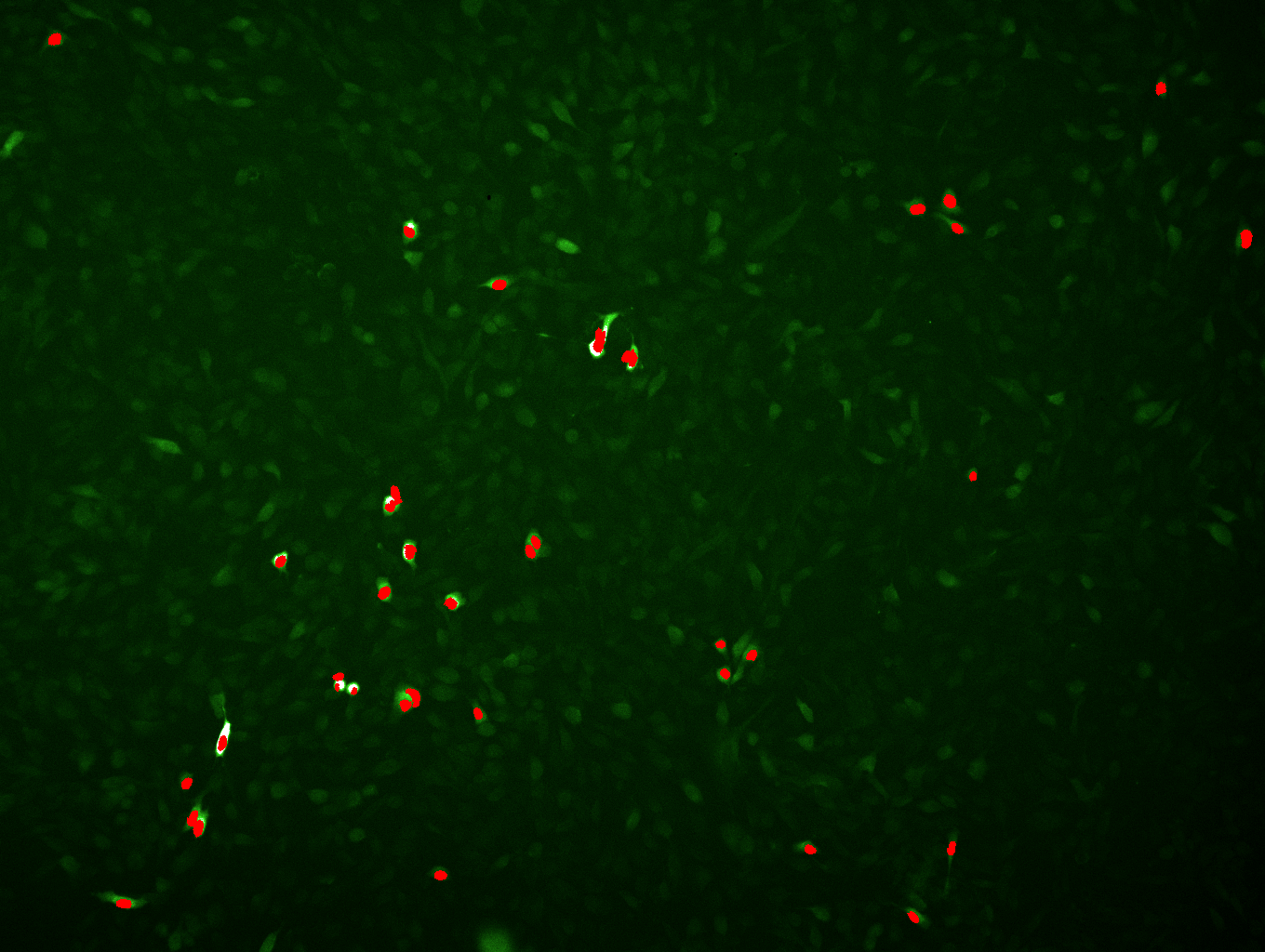

Supplement: Supplementary file 8 — Source Data for Figure 1 [file EMMM-11-e9930-s006.zip › Fig_1/Fig1E_ATG5_Pool_ROI.png]

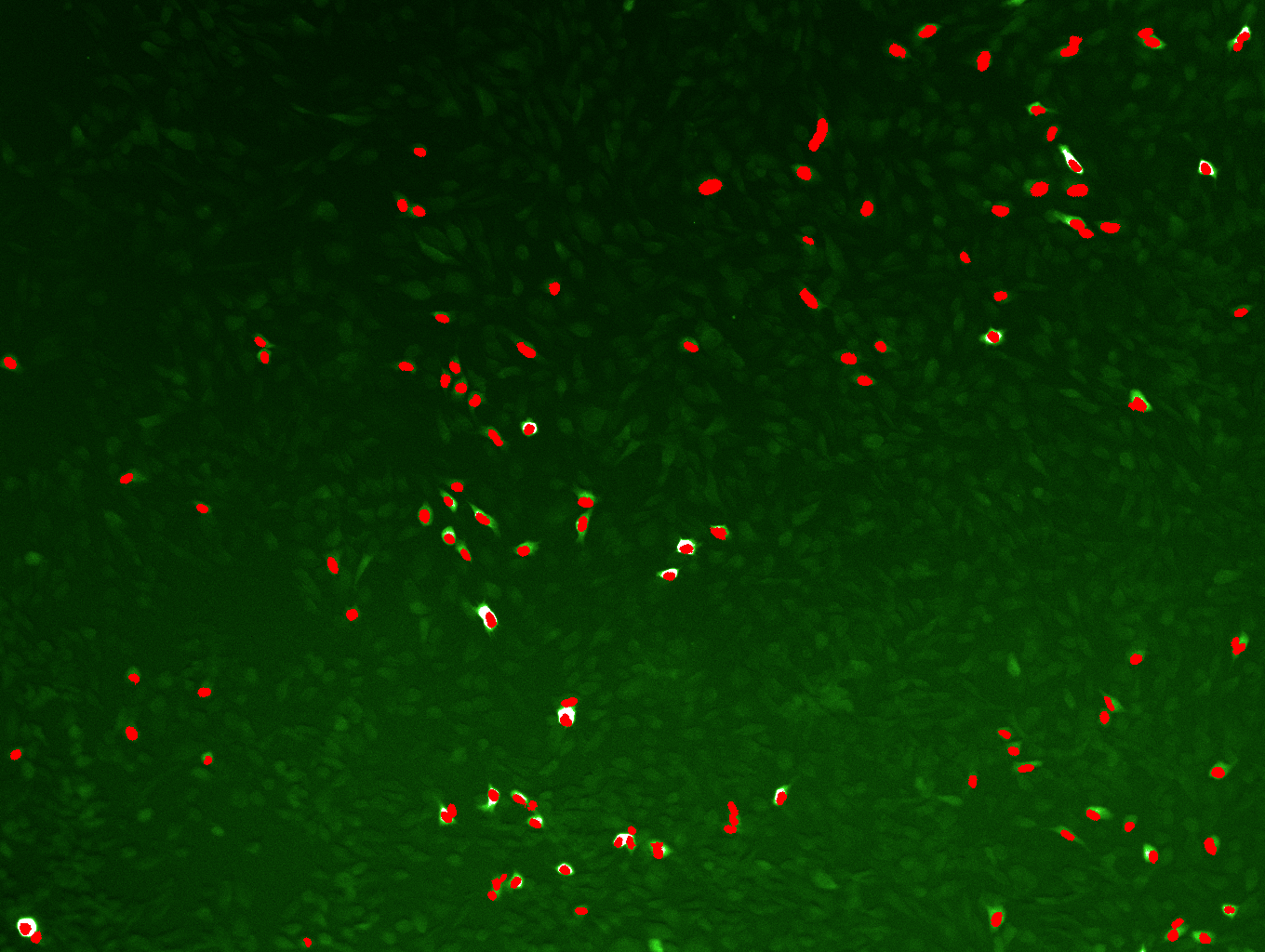

Supplement: Supplementary file 8 — Source Data for Figure 1 [file EMMM-11-e9930-s006.zip › Fig_1/Fig1E_EP300_Pool_ROI.png]

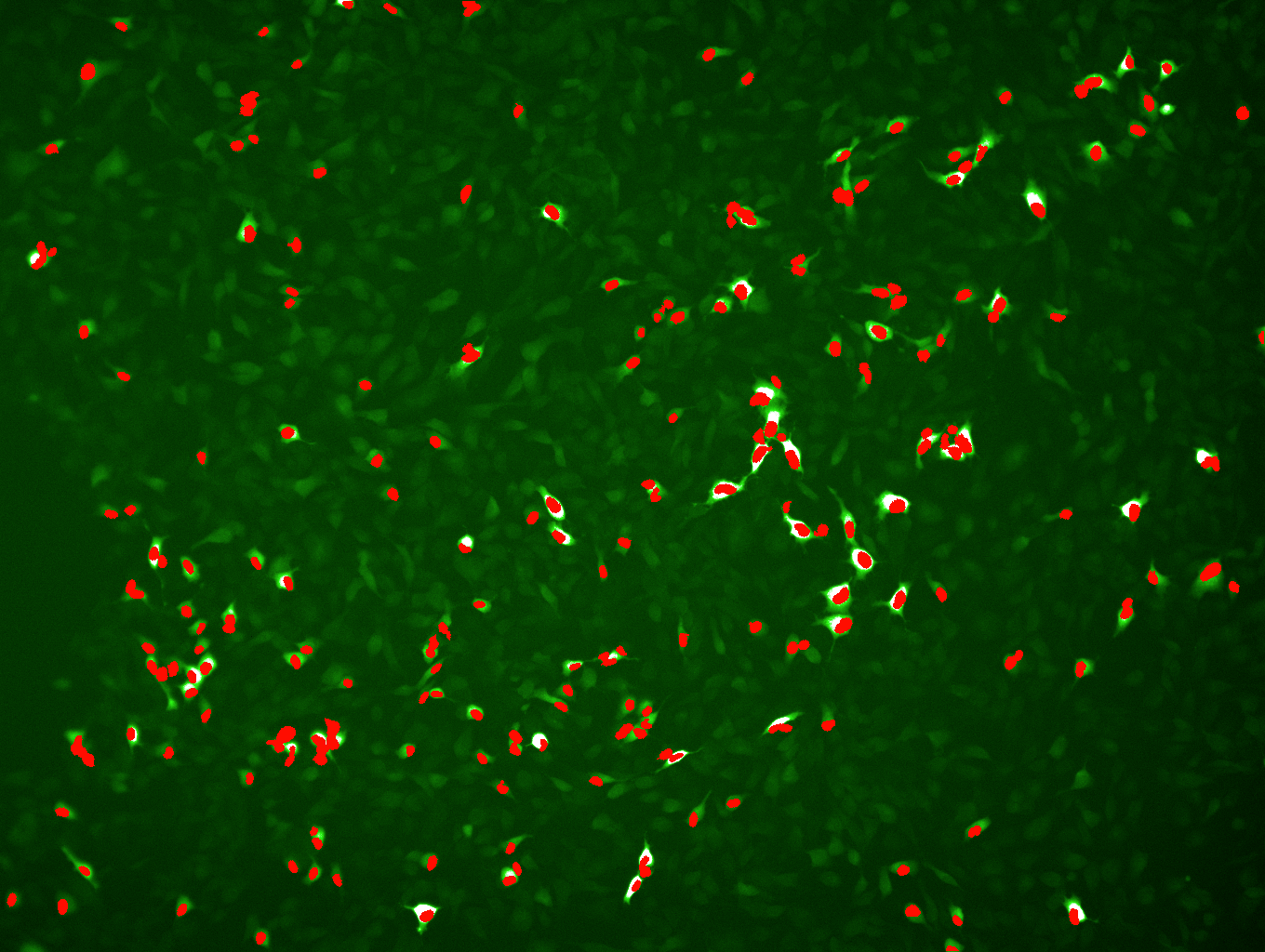

Supplement: Supplementary file 8 — Source Data for Figure 1 [file EMMM-11-e9930-s006.zip › Fig_1/Fig1E_EZH2_Pool_ROI.png]

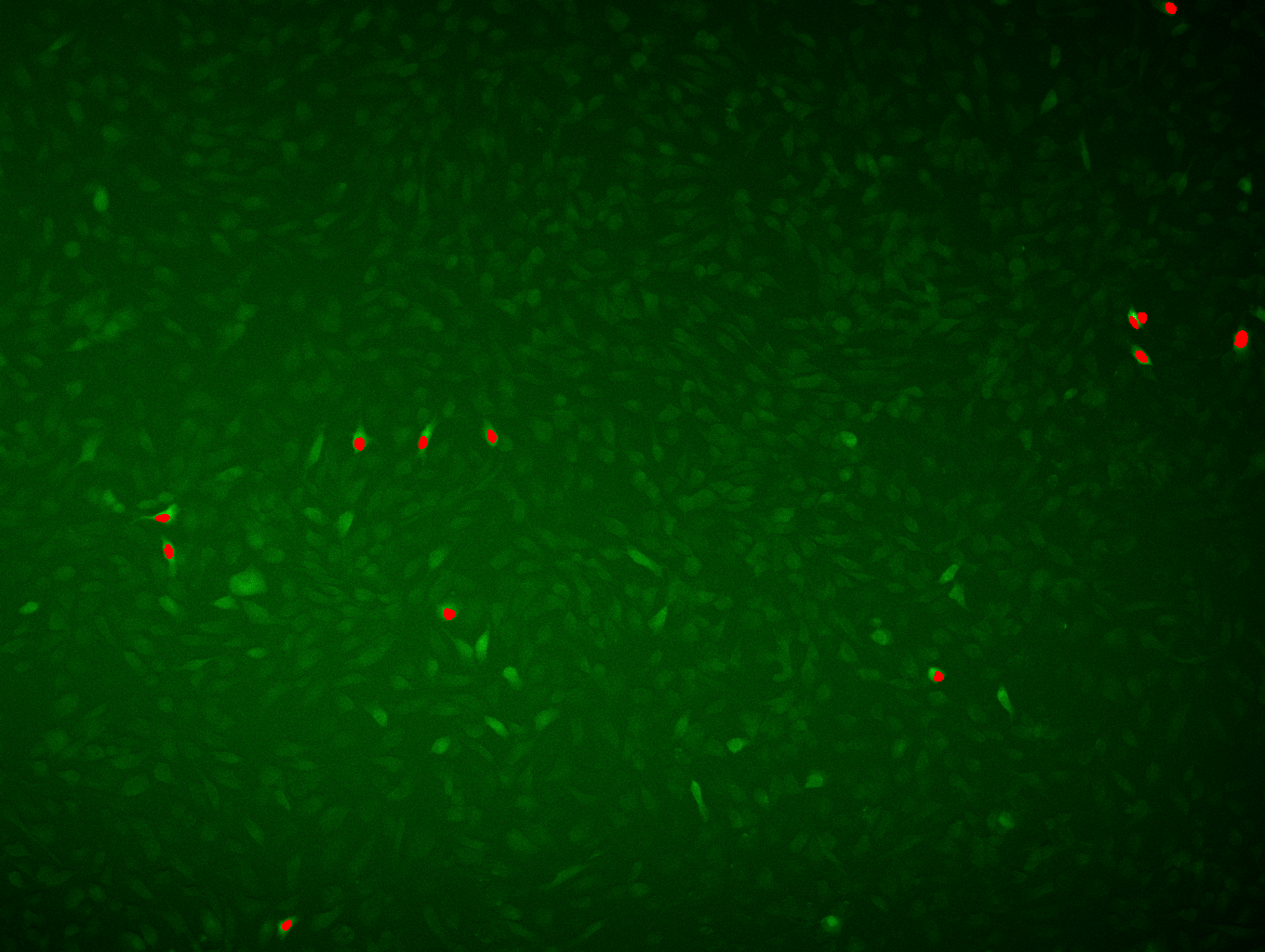

Supplement: Supplementary file 8 — Source Data for Figure 1 [file EMMM-11-e9930-s006.zip › Fig_1/Fig1E_MED9_siRNA1_ROI.png]

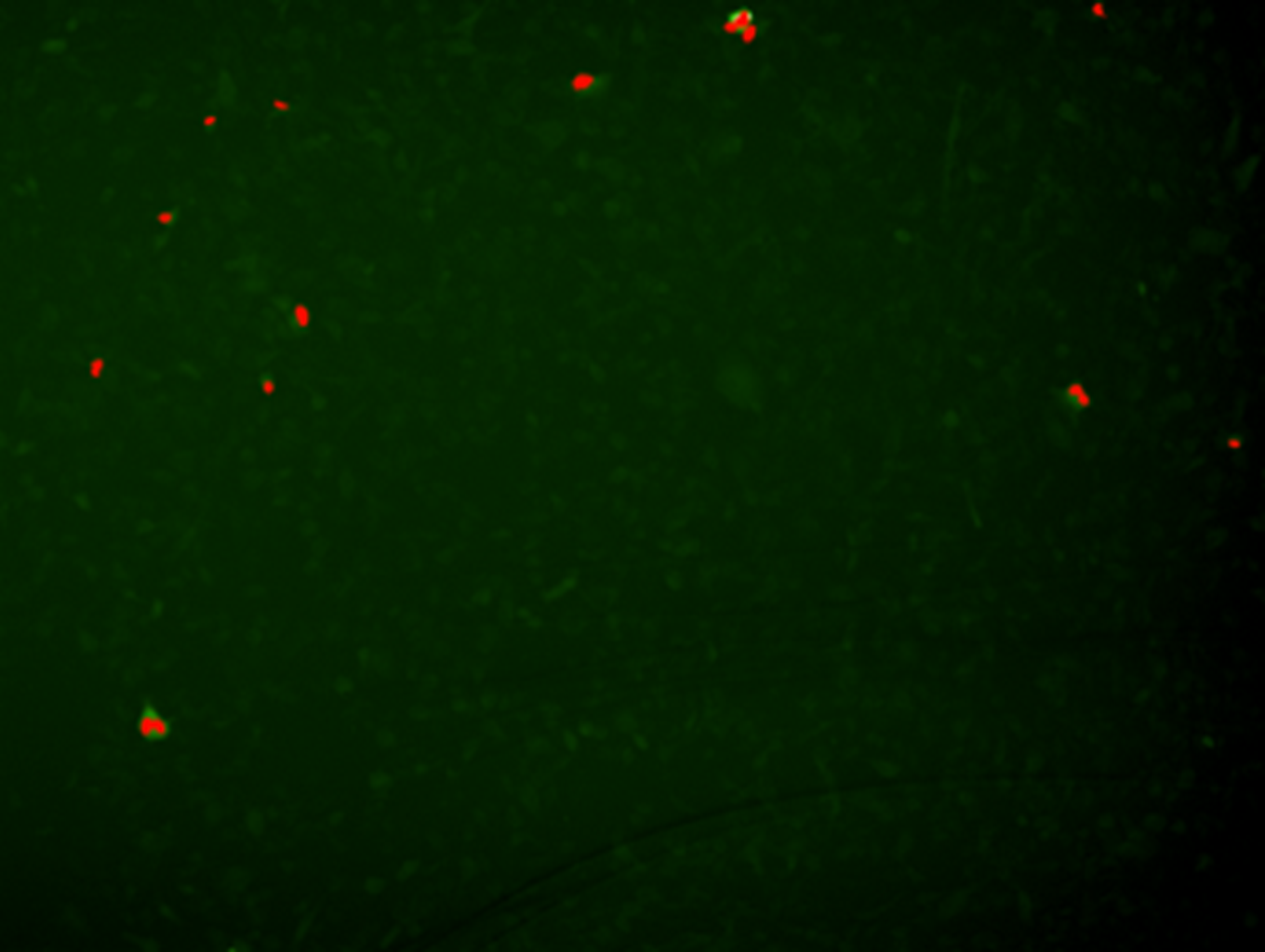

Supplement: Supplementary file 8 — Source Data for Figure 1 [file EMMM-11-e9930-s006.zip › Fig_1/Fig1E_NR3C1_Pool_ROI.png]

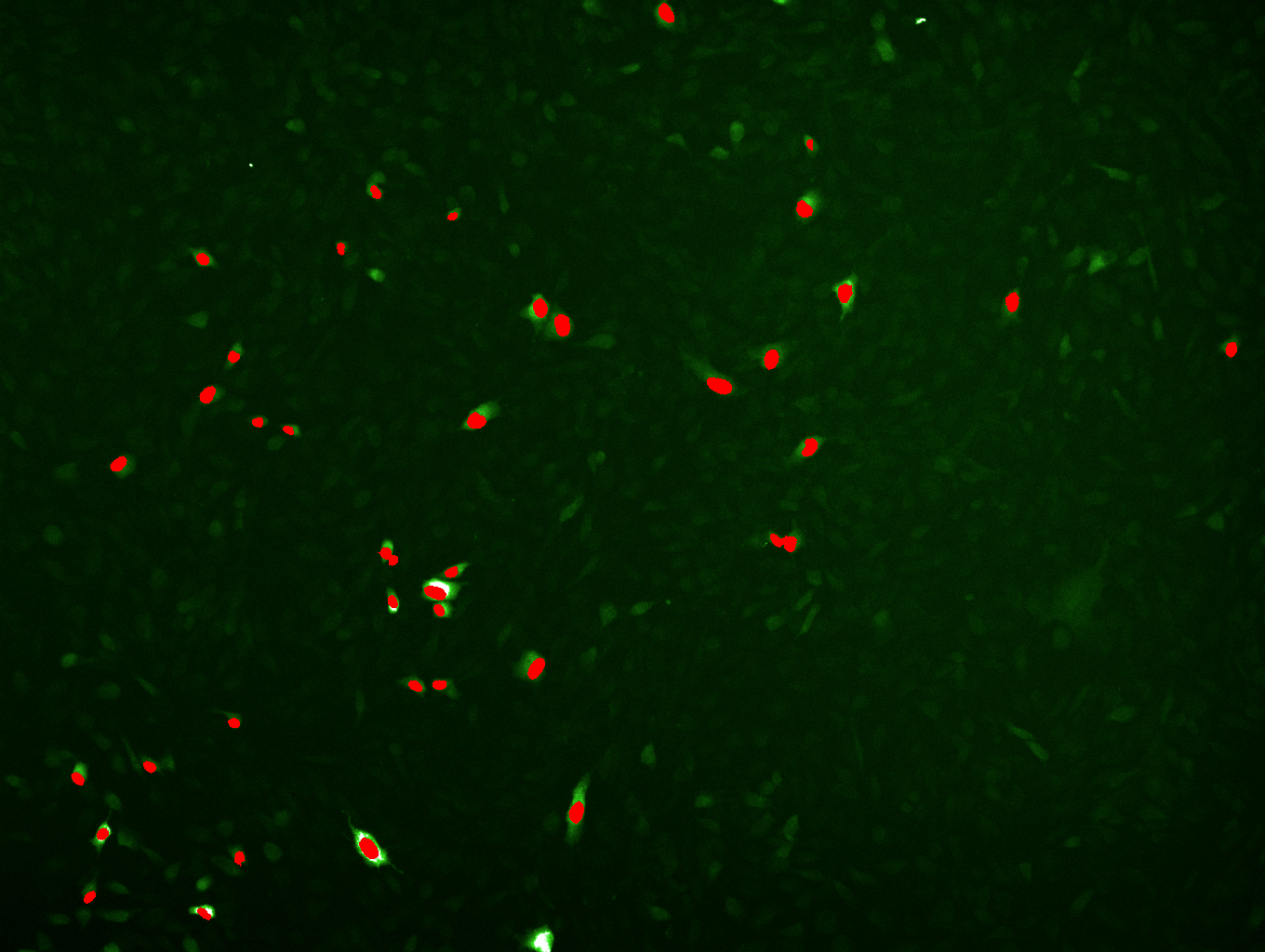

Supplement: Supplementary file 8 — Source Data for Figure 1 [file EMMM-11-e9930-s006.zip › Fig_1/Fig1E_Scramble_ROI.png]

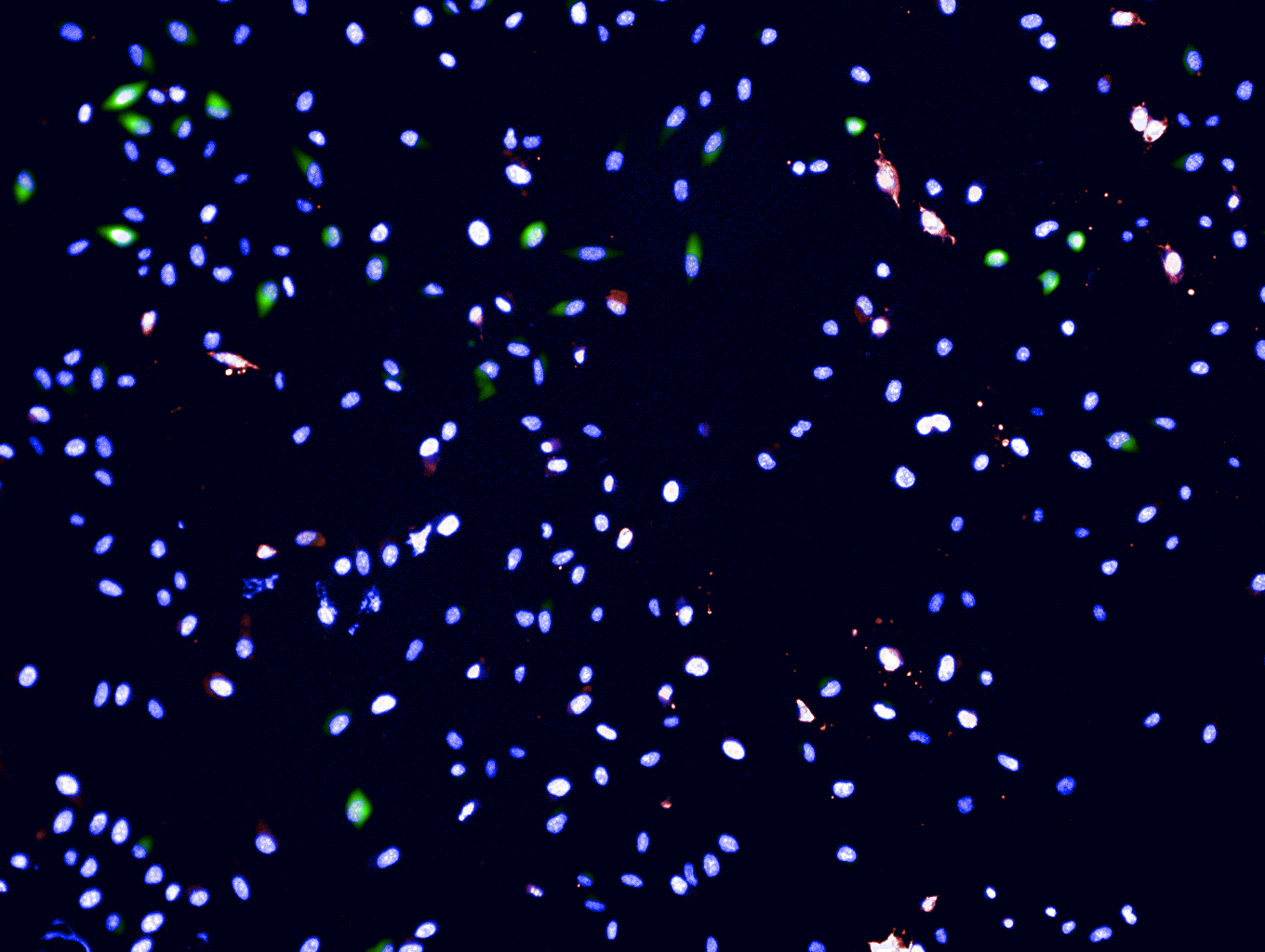

Supplement: Supplementary file 9 — Source Data for Figure 4 [file EMMM-11-e9930-s007.zip › Source_Data_for_Fig4/Fid4E_CisPt.png]

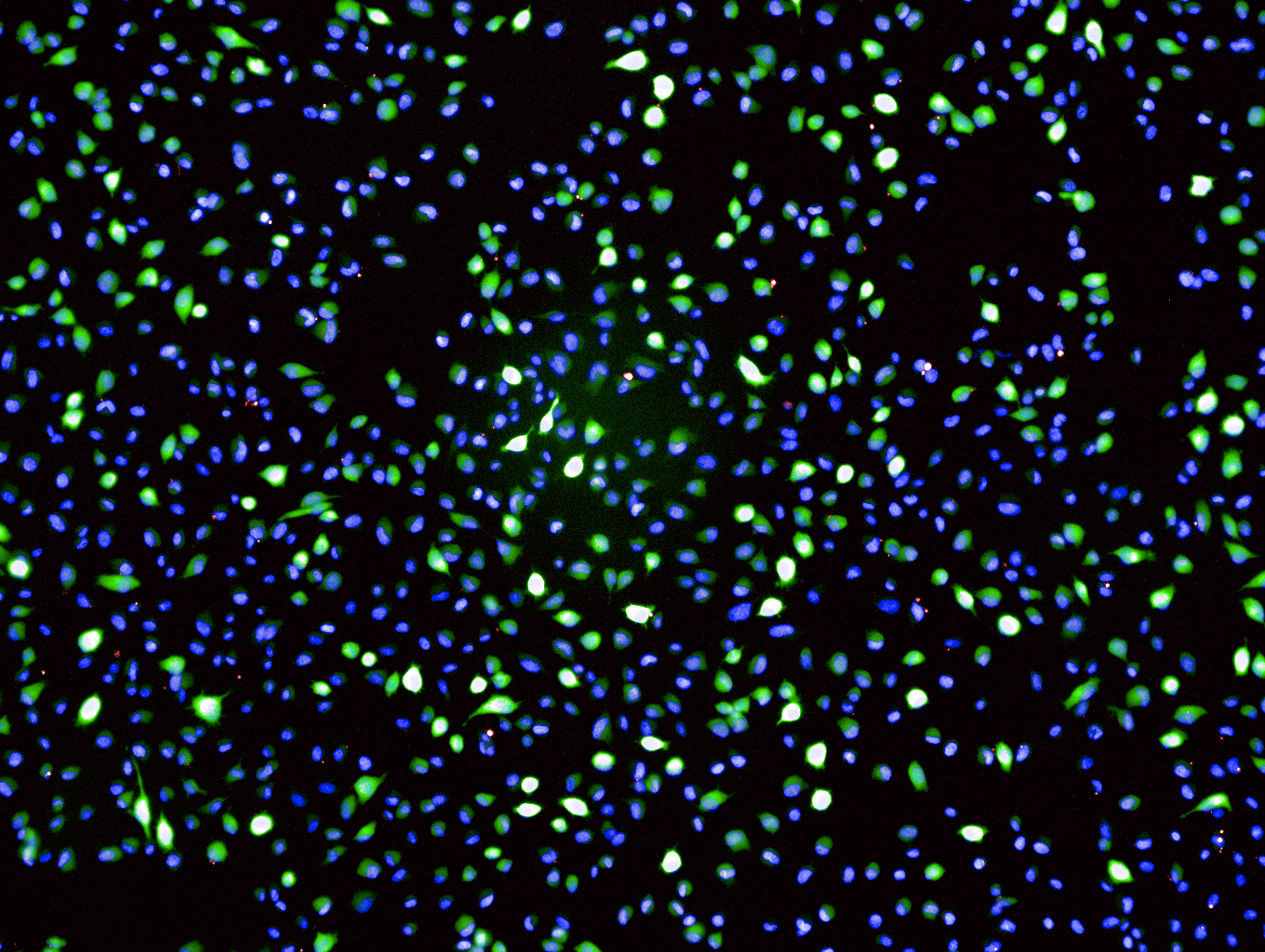

Supplement: Supplementary file 9 — Source Data for Figure 4 [file EMMM-11-e9930-s007.zip › Source_Data_for_Fig4/Fig4E_CTRL.png]

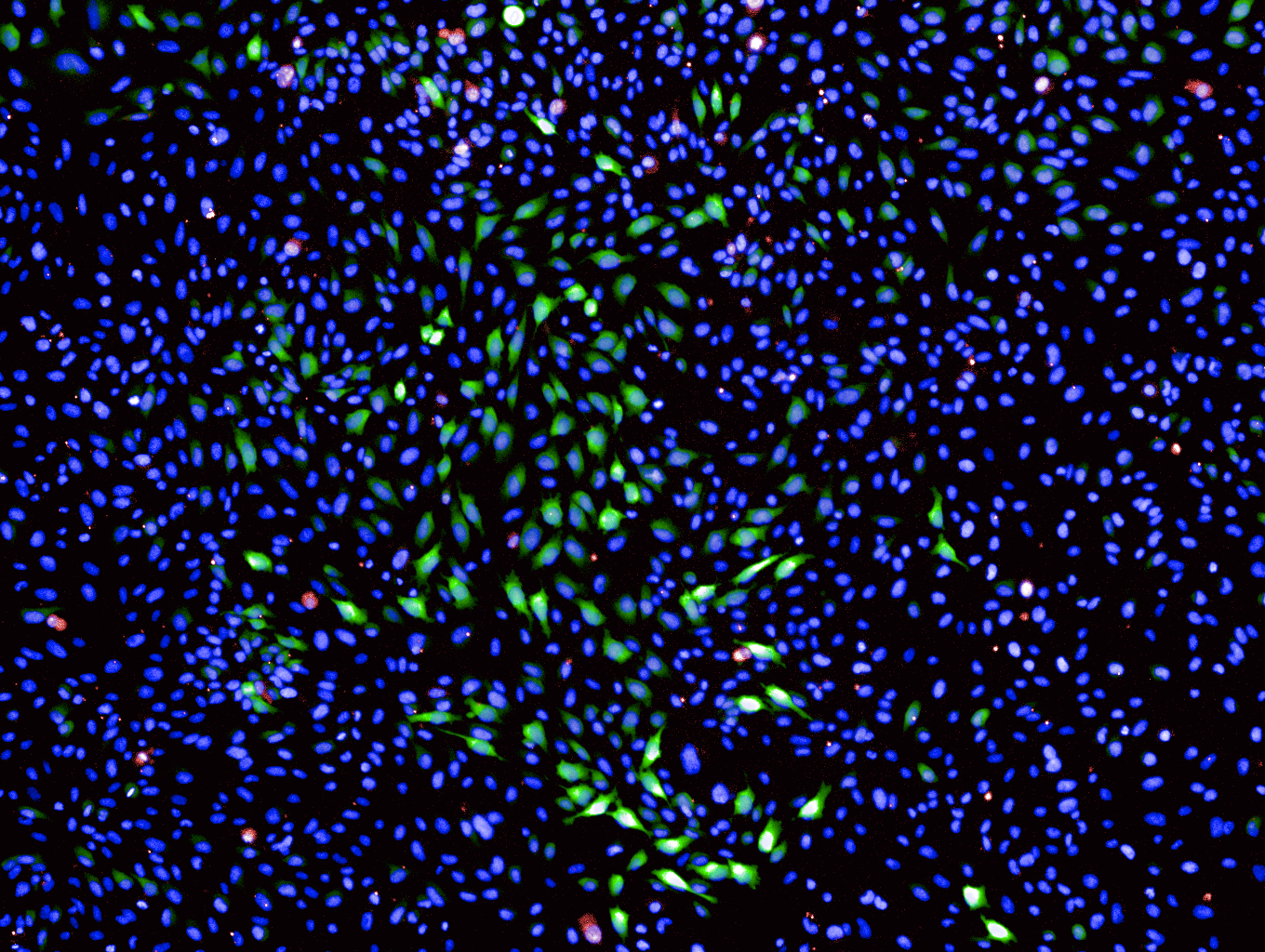

Supplement: Supplementary file 9 — Source Data for Figure 4 [file EMMM-11-e9930-s007.zip › Source_Data_for_Fig4/Fig_4E_SALJQ1.png]
